# Engineering Nitrogen Fixation Activity in an Oxygenic Phototroph

**DOI:** 10.1128/mBio.01029-18

**Published:** 2018-06-05

**Authors:** Deng Liu, Michelle Liberton, Jingjie Yu, Himadri B. Pakrasi, Maitrayee Bhattacharyya-Pakrasi

**Affiliations:** a Department of Biology, Washington University, St. Louis, Missouri, USA; University of Delaware

**Keywords:** cyanobacteria, N2 fixation, O2 tolerance, photosynthesis, synechocystis

## Abstract

Biological nitrogen fixation is catalyzed by nitrogenase, a complex metalloenzyme found only in prokaryotes. N_2_ fixation is energetically highly expensive, and an energy-generating process such as photosynthesis can meet the energy demand of N_2_ fixation. However, synthesis and expression of nitrogenase are exquisitely sensitive to the presence of oxygen. Thus, engineering nitrogen fixation activity in photosynthetic organisms that produce oxygen is challenging. Cyanobacteria are oxygenic photosynthetic prokaryotes, and some of them also fix N_2_. Here, we demonstrate a feasible way to engineer nitrogenase activity in the nondiazotrophic cyanobacterium *Synechocystis* sp. PCC 6803 through the transfer of 35 nitrogen fixation (*nif*) genes from the diazotrophic cyanobacterium *Cyanothece* sp. ATCC 51142. In addition, we have identified the minimal *nif* cluster required for such activity in *Synechocystis* 6803. Moreover, nitrogenase activity was significantly improved by increasing the expression levels of *nif* genes. Importantly, the O_2_ tolerance of nitrogenase was enhanced by introduction of uptake hydrogenase genes, showing this to be a functional way to improve nitrogenase enzyme activity under micro-oxic conditions. To date, our efforts have resulted in engineered *Synechocystis* 6803 strains that, remarkably, have more than 30% of the N_2_ fixation activity of *Cyanothece* 51142, the highest such activity established in any nondiazotrophic oxygenic photosynthetic organism. This report establishes a baseline for the ultimate goal of engineering nitrogen fixation ability in crop plants.

## IMPORTANCE

Application of chemically synthesized nitrogen fertilizers has revolutionized agriculture. However, the energetic costs of such production processes and the widespread application of fertilizers have raised serious environmental issues. A sustainable alternative is to endow to crop plants the ability to fix atmospheric N_2_ *in situ*. One long-term approach is to transfer all *nif* genes from a prokaryote to plant cells and to express nitrogenase in an energy-producing organelle, chloroplast, or mitochondrion. In this context, *Synechocystis* 6803, the nondiazotrophic cyanobacterium utilized in this study, provides a model chassis for rapid investigation of the necessary requirements to establish diazotrophy in an oxygenic phototroph.

## INTRODUCTION

The ability to introduce into crop plants the machinery to fix their own nitrogen via direct transfer of nitrogen fixation (*nif*) genes is envisioned to be key for the next agricultural revolution (1–3). However, engineering diazotrophic plants, however attractive a proposition, is an extreme challenge, due to the complexities in the biosynthesis of active nitrogenase, the enzyme that catalyzes nitrogen fixation, and the difficulty of coupling plant metabolism to supply energy and reducing power for the nitrogen fixation process (4). An additional impediment in the scenario is that photosynthesis produces O_2_, which is highly toxic with respect to the synthesis and activity of nitrogenase (5).

Diazotrophy occurs only in limited species of bacteria and archaea (6). Nitrogen fixation is mainly catalyzed by an iron- and molybdenum-dependent nitrogenase enzyme complex, with two enzymatic components, an iron protein dinitrogenase reductase (NifH) and an iron-molybdenum protein dinitrogenase (NifDK) (7, 8). Three metal-dependent cofactors, the F cluster, P cluster, and M cluster, are necessary to form the holoenzyme for electron transfer to reduce atmospheric N_2_ to form ammonia, the biologically available form of N_2_ (9, 10). A significant number of additional *nif* genes are required for the biosynthesis of these metallocluster cofactors and for the maturation of nitrogenase to form a fully functional enzyme (11, 12).

Transferring nitrogen fixation to nondiazotrophs has been attempted for decades. To date, the heterotrophic bacterium Escherichia coli has been successfully engineered for nitrogen fixation activity through transfer of *nif* genes from various diazotrophic species (13–17). Attempts to engineer eukaryotic species for heterologous nitrogen fixation activity, including the yeast Saccharomyces cerevisiae and the green alga Chlamydomonas reinhardtii, have been unsuccessful. Limited success was reached only in expressing the NifH component as an active moiety in Chlamydomonas reinhardtii (18). While all the Nif components have been successfully expressed in yeast cells, the formation of a fully functional nitrogenase complex has not yet been achieved (19–22).

Expression of nitrogenase components in plants has also been attempted in a few studies. Individual expression of 16 Nif proteins targeted to the plant mitochondria has been reported recently, but none of the structural components showed enzymatic activity (23). Another recent study showed that an active NifH component can be formed in tobacco chloroplasts (24), indicating that expression of active nitrogenase in chloroplasts might be a viable way to engineer crop plants to fix nitrogen in the future (25). Since it is widely accepted that a cyanobacterial ancestor was the progenitor of chloroplasts (26), engineering a cyanobacterium to fix nitrogen may pave the way to achieving the final goal of engineering nitrogen-fixing ability into crop plants. We have utilized the nondiazotrophic cyanobacterium *Synechocystis* sp. PCC 6803 (here *Synechocystis* 6803) as a chassis to engineer nitrogen fixation activity into an oxygenic photosynthetic organism.

**FIG 1:**
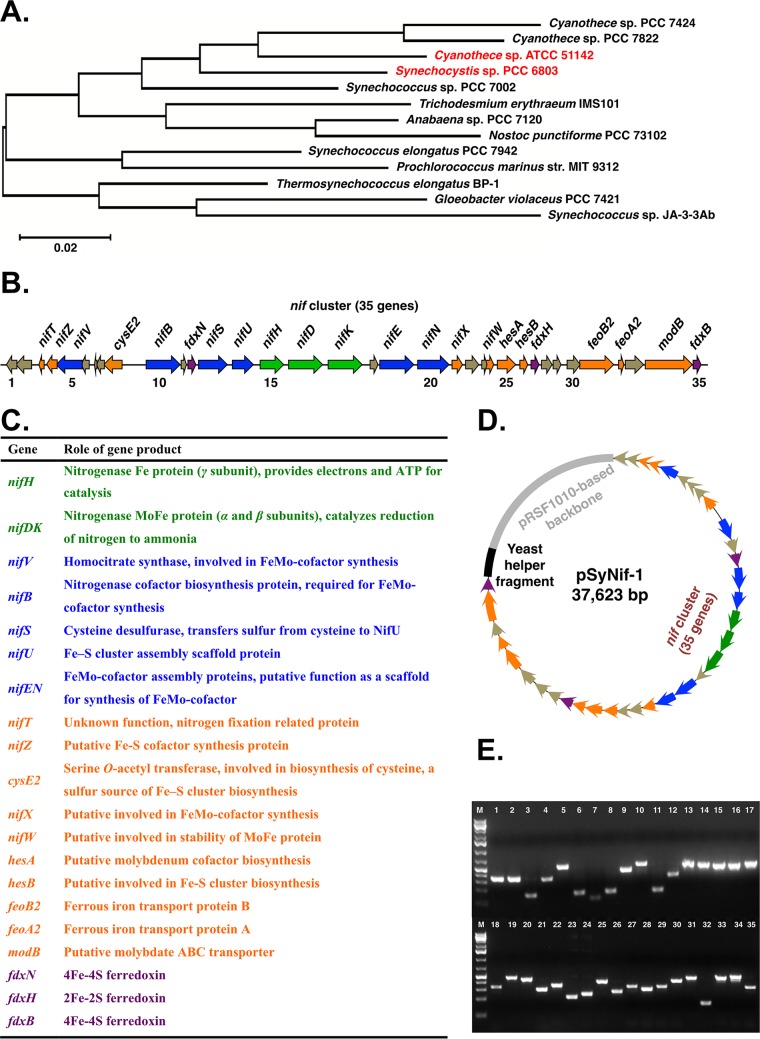
Introduction of *nif* genes into *Synechocystis* 6803. (A) Maximum likelihood 16S rRNA gene phylogeny of cyanobacteria. (B and C) Schemes showing the genetic organization of the *nif* cluster (B) and the role of each gene product (C) in *Cyanothece* 51142. Shown are the genes for the three structural proteins (*nifHDK*; green), necessary cofactors (blue), accessory proteins (orange), ferredoxins (purple), and hypothetical proteins (brown). Gene names and annotations are from GenBank (https://www.ncbi.nlm.nih.gov/genbank/) and Cyanobase (http://genome.microbedb.jp/cyanobase). (D) A schematic map of plasmid pSyNif-1 containing the entire *nif* cluster. The backbone (gray) is from broad host plasmid pRSF1010, which can replicate in *Synechocystis* 6803. The yeast helper fragment (black) contains *CEN6* and *ARS* as an *ori* and *ura3* as a selection marker. (E) Transcription of all 35 genes in engineered *Synechocystis* 6803. Each lane represents a gene in the *nif* cluster, as numbered in panel B. Total RNA was extracted from cells cultured in BG11_0_ medium under 12-h light/12-h dark conditions, and cDNA was used as the template for PCR.

The unicellular diazotrophic cyanobacterium *Cyanothece* sp. ATCC 51142 (here *Cyanothece* 51142) uses temporal separation as its strategy to protect nitrogenase from O_2_ produced by photosynthesis (27, 28). Within *Cyanothece*, the two conflicting processes, photosynthesis and N_2_ fixation, occur sequentially during the diurnal periods, such that photosynthesis and O_2_ evolution are performed during the day whereas N_2_ is fixed at night (29). The energy requirements for nitrogenase are met in *Cyanothece* by the catabolism of glycogen. Glycogen is accumulated in the light as the storage form of fixed CO_2_ and is later degraded in the dark to provide energy for nitrogenase. The provision of energy coupled with high rates of respiration ensures a low-oxygen intracellular environment and sufficient supplies of energy for N_2_ fixation (30). The *Cyanothece* 51142 genome contains the most complete contiguous set of nitrogen fixation and related genes forming the *nif* cluster, which contains 35 genes (cce_0545 to cce_0579), encoding structural proteins, metal cofactor synthesis proteins, ferredoxins, and proteins with unknown but necessary functions (Fig. 1). All 35 genes exhibit similar oscillating diurnal patterns of transcription during light/dark cycles, showing a high level of transcription in the dark and notably reduced levels in light (27). Such synchronized transcriptional patterns also confirm that all of these genes are related to nitrogen fixation.

In the current study, we successfully transferred and expressed this large *nif* gene cluster in nondiazotrophic *Synechocystis* 6803 with resultant N_2_ fixation activity. Subsequent engineering of the cluster and its expression levels have led to nitrogenase activities as high as 30% of that in *Cyanothece* 51142.

## RESULTS AND DISCUSSION

### Introduction of *nif* genes into *Synechocystis* 6803.

**FIG 2:**
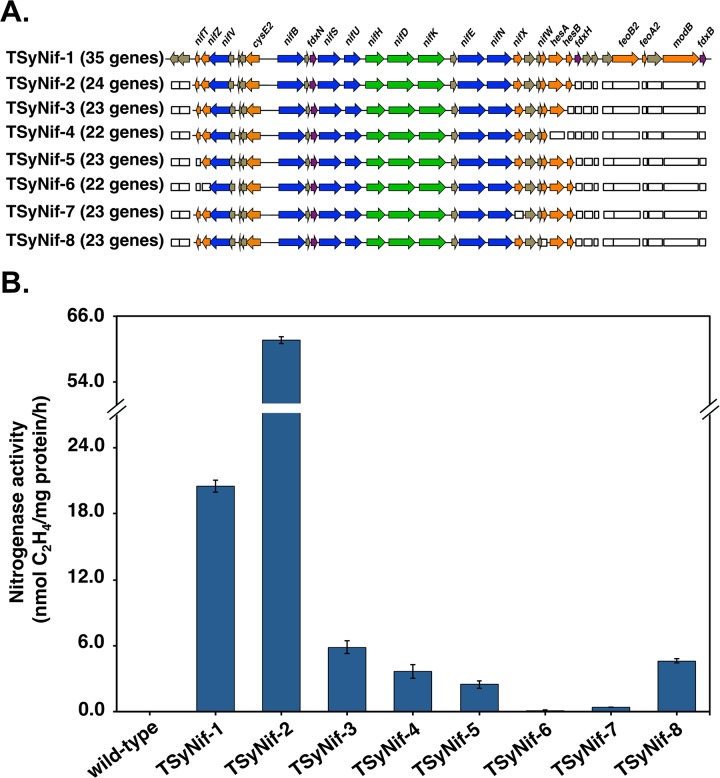
The minimal *nif* cluster required for nitrogen fixation activity in *Synechocystis* 6803. (A) Scheme showing the top-down method to determine the minimal *nif* cluster. The hollow rectangles represent the genes deleted from the cluster, and the colored rectangles represent the remaining genes. (B) Nitrogen fixation activity in engineered strains. Samples were collected from cultures under 12-h light/12-h dark conditions in BG11_0_ medium. Nitrogen fixation activity was assayed by acetylene reduction, and error bars represent the standard deviations observed from at least three independent experiments.

*Synechocystis* 6803 has a close phylogenetic relationship with *Cyanothece* 51142 (Fig. 1A) (31). The large *nif* cluster from *Cyanothece* 51142 (28.34-kb region of DNA) was transferred into wild-type *Synechocystis* 6803 on a single extrachromosomal plasmid. This large plasmid, pSyNif-1, containing the entire *nif* cluster with 35 genes (Fig. 1D), was constructed using the DNA assembler method (32). The chassis of this vector was based on pRSF1010 (33), and this self-replicating pSyNif-1 plasmid was transferred into *Synechocystis* 6803 by conjugation, generating the engineered strain TSyNif-1. Remarkably, over the past 4 years since its introduction into the heterologous host, pSyNif-1 has been stably maintained in its entirety in *Synechocystis* (see Fig. S1 in the supplemental material). Furthermore, all introduced *Cyanothece* genes were transcribed (Fig. 1E) as detected by reverse transcription-PCR (RT-PCR), indicating that native promoters in the *nif* cluster from *Cyanothece* 51142 can drive transcription of genes in *Synechocystis* 6803. An acetylene reduction assay method for nitrogen fixation detected nitrogenase activity under 12-h light/12-h dark conditions for strain TSyNif-1 (Fig. 2). Nitrogen fixation reached an activity level of 2% relative to that in *Cyanothece* 51142 grown under similar conditions. This is the first time that a nondiazotrophic phototroph has been engineered for biosynthesis of a fully functional nitrogenase enzyme and has been found to exhibit detectible and stable nitrogen fixation activity.

10.1128/mBio.01029-18.1 FIG S1  Growth of engineered strains compared to the wild-type strain of *Synechocystis* 6803. Cells were cultured in BG11 medium under 12-h light/12-h dark conditions. Error bars represent the standard deviations of results from at least three independent experiments. Download FIG S1, PDF file, 0.05 MB. Copyright © 2018 Liu et al. 2018 Liu et al. This content is distributed under the terms of the Creative Commons Attribution 4.0 International license.

### The minimal required gene cluster for nitrogen fixation activity.

**FIG 3:**
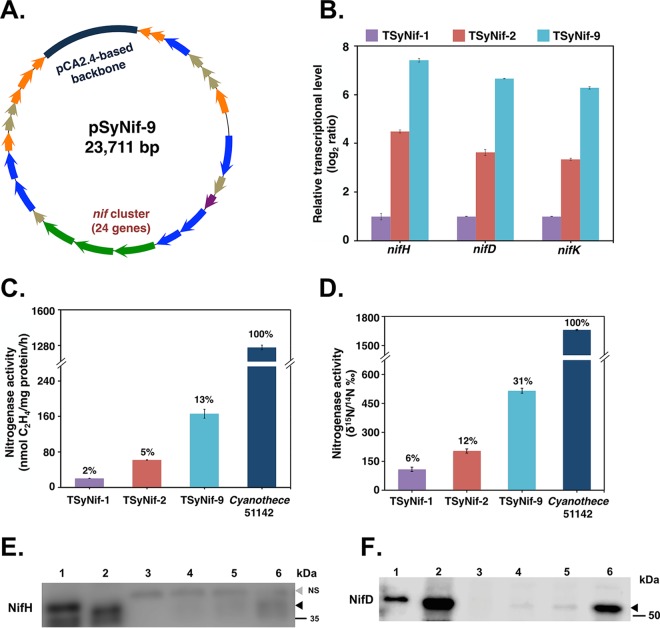
Enhancement of transcription levels of *nif* genes leads to higher nitrogen fixation activity. (A) A schematic map of plasmid pSyNif-9 containing the *nif* cluster with 24 genes from *nifT* to *hesB*. The backbone (dark blue) is from endogenous plasmid pCA2.4 of *Synechocystis* 6803. (B) Comparison of transcription levels of the *nif* structural genes in engineered strains through quantitative PCR (q-PCR). (C and D) Comparison of nitrogen fixation activities in engineered strains, as measured by C_2_H_2_ reduction assay (C) as well as ^15^N assimilation assay (D). (E) Western blot showing the presence of NifH protein in engineered *Synechocystis* 6803 strains. Lanes 1 to 6 represent 2.0 µg purified NifH-His protein from E. coli and 15 µg whole-cell extracts of *Cyanothece* 51142, the *Synechocystis* 6803 wild-type strain, TSyNif-1, TSyNif-2, and TSyNif-9, respectively. The black triangle indicates the band for NifH protein, while the gray one indicates the nonspecific band. (F) Western blot showing the presence of NifD protein in engineered *Synechocystis* 6803 strains. Lanes 1 to 6 represent 0.5 µg purified NifD-His protein from E. coli and 15 µg whole-cell extracts of *Cyanothece* 51142, the *Synechocystis* 6803 wild-type strain, TSyNif-1, TSyNif-2, and TSyNif-9, respectively. Cyanobacterial samples were collected from cultures under 12-h light/12-h dark conditions in BG11_0_ medium. Error bars represent the standard deviations of results from at least three independent experiments.

Gene expression parameters for *Synechocystis* 6803 are not as well understood as those for E. coli. Thus, the refactoring of *nif* genes as performed in E. coli and *Klebsiella* (16, 34) to determine the minimal requirement of genes for nitrogen fixation in *Synechocystis* 6803 is impractical at this stage. Therefore, we approached the identification of a minimal *nif* cluster for nitrogen fixation using a “top-down” method, which determines the influence of a gene on nitrogenase activity by selectively removing individual genes from the *nif* cluster (Fig. 2). Extrapolating the genetic requirements for nitrogen fixation activity observed in studies in which *nif* genes were introduced in E. coli (14, 15), we determined that genes for all homologous proteins introduced into E. coli are present in the *Cyanothece* 51142 *nif* cluster between gene *nifT* and *hesB* (Fig. 1). Hence, our second plasmid, pSyNif-2, contains 24 genes in the *nif* cluster between *nifT* and *hesB* (Fig. 2). Eleven genes were removed that presumably encode three metal transporter proteins, two ferredoxins, and six proteins of unknown function, none of which have been analyzed previously, although they are associated with nitrogen fixation. Intriguingly, this second engineered TSyNif-2 strain with a reduced cluster of 24 genes has a 3-fold increase in nitrogen fixation activity compared to strain TSyNif-1 (Fig. 2). Although plasmids pSyNif-1 and pSyNif-2 have the same plasmid backbone (Fig. S2), the transcriptional levels of the structural genes *nifH*, *nifD*, and *nifK* are higher in the TSyNif-2 strain (Fig. 3). This improvement in nitrogenase activity could be the result of removal of one or more regulatory genes, which may encode a protein(s) that represses expression of genes in the *nif* cluster.

10.1128/mBio.01029-18.2 FIG S2  Schematic map of plasmids used for expression of *nif* genes in *Synechocystis* sp. PCC 6803. Replicating plasmids (A) pSyNif-1 (pSL2379) and (B) pSyNif-2 (pSL2397) were constructed using the DNA assembler method and introduced into the wild-type strain of *Synechocystis* 6803 by conjugation. The components of the plasmids are labeled as follows: backbone containing the bacterial replication machinery (gray), yeast helper fragment containing *CEN6* and *ARS H4* as an *ori* and containing *ura3* as a selection marker (black), and the *nif* gene cluster from *Cyanothece* ATCC 51142. The colors of the *nif* genes are the same as in Fig. 1. Download FIG S2, PDF file, 0.3 MB. Copyright © 2018 Liu et al. 2018 Liu et al. This content is distributed under the terms of the Creative Commons Attribution 4.0 International license.

However, further removal of genes from both directions resulted in a decrease of nitrogen fixation activity by more than 10-fold for strains TSyNif-3 to TSyNif-6, from which genes *hesB*, *hesAB*, *nifT*, and *nifTZ* were removed, respectively (Fig. 2). Thus, this “top-down” approach determined that an essential minimal cluster from *nifT* to *hesB* is required for nitrogen fixation activity in *Synechocystis* 6803. Additionally, we investigated the removal of two more genes in the cluster, *nifX* and *nifW*, generating the two strains TSyNif-7 and TSyNif-8. Deletion of these two genes did not affect expression of surrounding genes (Fig. S3). However, nitrogen fixation activity levels dropped 100-fold and 10-fold, respectively, in these engineered strains (Fig. 2). We conclude that both *nifX* and *nifW* are important genes for nitrogen fixation. Notably, *nifX* exhibited a positive influence on N_2_ fixation in cyanobacteria, while it functions as a negative regulator for N_2_ fixation in the heterotrophic diazotroph Klebsiella oxytoca (35).

10.1128/mBio.01029-18.3 FIG S3  Transcription of *nif* genes in engineered *Synechocystis* 6803. (A) Transcripts of *nif* genes in *Synechocystis* 6803 strain TSyNif-2. Gene expression was detected by RT-PCR. Lanes 1 to 24 show genes from *nifT* to *hesB* in the cluster (Fig. 1B). (B to G) Expression of *nifHDK* and surrounding genes was examined in each engineered strain shown in Fig. 2 by RT-PCR. (B) TSyNif-3 (23 genes without *hesB* compared to TSyNIf-2). (C) TSyNif-4 (22 genes without *hesAB* compared to TSyNif-2). (D) TSyNif-5 (23 genes without *nifT* compared to TSyNif-2). (E) TSyNif-6 (22 genes without *nifTZ* compared to TSyNif-2). (F) TSyNif-7 (23 genes without *nifX* compared to TSyNif-2). (G) TSyNif-8 (23 genes without *nifW* compared to TSyNif-2). Cultures for RNA extraction were grown under 12-h light/12-h dark conditions in BG11_0_ medium. Download FIG S3, PDF file, 4.3 MB. Copyright © 2018 Liu et al. 2018 Liu et al. This content is distributed under the terms of the Creative Commons Attribution 4.0 International license.

### Improvement of nitrogen fixation activity.

To increase the RNA expression levels of the nitrogenase-related genes, we took advantage of three small endogenous plasmids in *Synechocystis* 6803: pCA2.4, pCB2.4, and pCC5.2. The heterologous genes harbored in these endogenous plasmids maintained higher transcriptional levels than those in a pRSF1010-based plasmid, because of the higher copy numbers of these three plasmids within *Synechocystis* (36, 37). First, we replaced the RSF1010 backbone of plasmid pSyNif-2 by the entire DNA segment of each of these endogenous episomes and then transferred the plasmids to *Synechocystis* 6803, generating three strains, TSyNif-9, TSyNif-10, and TSyNif-11, with the chassis of pCA2.4, pCB2.4, and pCC5.2, respectively (Fig. 3A; see also Fig. S4). As expected, genes *nifH*, *nifD*, and *nifK* in strain TSyNif-9 showed transcription levels that were severalfold higher than in TSyNif-2 (Fig. 3B). In addition, nitrogen fixation activity was increased by another 2- to 3-fold, reaching 13% for the acetylene reduction activity in TSyNif-9 relative to that observed in *Cyanothece* 51142 (Fig. 3C). Next, nitrogenase activity was directly assayed in the engineered strains using a ^15^N assimilation assay method. Remarkably, the highest activity obtained was from strain TSyNif-9, reaching 31% of ^15^N assimilation relative to *Cyanothece* 51142 (Fig. 3D). The activity data presented here is comparable to published data from studies on nitrogen fixation activity in engineered E. coli strains (Table 1). Additionally, both the NifH and NifD nitrogenase structural proteins were detected in whole-cell extracts via Western blotting by using antisera against the NifH and NifD proteins of Rhodospirillum rubrum, respectively (Fig. 3E and F). Although the protein level in *Cyanothece* 51142 is significantly higher than in the engineered *Synechocystis* 6803 strains, the NifD protein level in strain TSyNif-9 reached 10% of total cellular proteins (Fig. S4). It was also evident that the nitrogenase activities in the engineered strains were proportional to the level of nitrogenase structural proteins, which implied that optimizing the expression of nitrogenase proteins is critical for the activity. Most importantly, from an evolutionary standpoint, these results highlight the potential for engineering plant chloroplasts to fix nitrogen at a high level of activity, since oxygenic cyanobacteria are the progenitors of chloroplasts.

10.1128/mBio.01029-18.4 FIG S4  Improvement of nitrogen fixation activity by increasing plasmid copy number. (A and B) Schematic maps of plasmid pSyNif-10 and plasmid pSyNif-11 containing the *nif* cluster with 24 genes from *nifT* to *hesB*. The backbone (dark blue) is from endogenous plasmid pCB2.4 and plasmid pCC5.2 of *Synechocystis* 6803, respectively. (C) Comparison of nitrogen fixation activities in engineered strains measured by ^15^N assimilation assay. (D) Western blot showing the presence of the NifD protein in engineered *Synechocystis* 6803 cells. Lanes 1 to 3 represent 1.0 µg, 0.5 µg, and 0.25 µg purified NifD-His protein from E. coli, respectively; lanes 4 to 9 represent 15 µg whole-cell extracts of TSyNif-9, TSyNif-10, TSyNif-11, TSyNif-1, TSyNif-2, and the *Synechocystis* 6803 wild-type strain, respectively. Samples were collected from cultures grown under 12-h light/12-h dark conditions in BG11_0_ medium. Error bars represent the standard deviations of results from at least three independent experiments. Download FIG S4, PDF file, 0.5 MB. Copyright © 2018 Liu et al. 2018 Liu et al. This content is distributed under the terms of the Creative Commons Attribution 4.0 International license.

**TABLE 1:** Nitrogen fixation activity in diazotrophs and engineered strains

Strain	Nitrogenase activity (nmol C_2_H_4_/mg protein/h)	% activity (based on C_2_H_2_ reduction assay)	% activity (based on ^15^N assimilation assay)	Source or reference
Diazotrophs				
*Cyanothece* 51142	1,262	100	100	This study
Azotobacter vinelandii	3,300	100	100	52
*Paenibacillus* sp. strain WLY78	3,050	100	100	14
Pseudomonas stutzeri A1501	1,050	100	ND^e^	53
Klebsiella oxytoca M5a1	3,708	100	ND	54
				
Engineered strains				
TSyNif-1	20	2	6	This study
TSyNif-9	166	13	31	This study
Engineered E. coli^a^	180	5	35	15
Engineered E. coli^b^	300	10	30	14
Engineered E. coli^c^	105	10	ND	53
Engineered E. coli^d^	740	20	ND	16

a Nitrogen fixation genes from A. vinelandii and K. oxytoca.

b Nitrogen fixation genes from *Paenibacillus*.

c Nitrogen fixation genes from *Pseudomonas*.

d Nitrogen fixation genes from K. oxytoca.

e ND, not determined.

### Nitrogen fixation activity in *Synechocystis* 6803.

Despite the additional metabolic load of expressing large cohorts of 35 or 24 genes related to nitrogen fixation being introduced in *Synechocystis* 6803, remarkably, the expression and activities of these heterologous proteins did not affect the growth of the engineered strains under diurnal light/dark conditions (Fig. S1). We used strain TSyNif-2 to assess the influence of oxygen and exogenous nitrate on nitrogenase activity under four conditions, BG11, BG11_0_ (BG11 without nitrate), BG11 with 10 µM DCMU [3-(3,4-dichlorophenyl)-1,1 dimethylurea] (no O_2_ evolution), and BG11_0_ with 10 µM DCMU (Fig. S5). Interestingly, the transcript levels of the *nifH*, *nifD*, and *nifK* genes in the TSyNif-2 strain were downregulated by nitrate, which is similar to the results seen with *Cyanothece* 51142. Specifically, the depletion of nitrate improved the nitrogenase activity over 30-fold in BG11_0_ with DCMU (Fig. S5). Nitrogen fixation activity was obtained only in an anaerobic environment when DCMU was added to the testing culture, although the headspace of all cultures was flushed with pure argon. These data indicate that oxygen generated by photosynthesis directly blocks nitrogenase activity in TSyNif-2, highlighting that one of the biggest challenges for engineering nitrogen fixation in oxygenic phototrophs is the sensitivity of nitrogenase to oxygen.

10.1128/mBio.01029-18.5 FIG S5  Characteristics of engineered *Synechocystis* 6803 strain TSyNif-2. (A) Comparison of nitrogenase activities in the TSyNif-2 strain under four different conditions. (B) Transcript levels of *nifH*, *nifD*, and *nifK* genes in TSyNif-2 strains were assayed by q-PCR. RNA was extracted from the cells grown under 12-h light/12-h dark conditions. Error bars represent the standard deviations of results from three independent replicates. Download FIG S5, PDF file, 0.1 MB. Copyright © 2018 Liu et al. 2018 Liu et al. This content is distributed under the terms of the Creative Commons Attribution 4.0 International license.

### Improvement of O_2_ tolerance by introduction of hydrogenase uptake.

**FIG 4:**
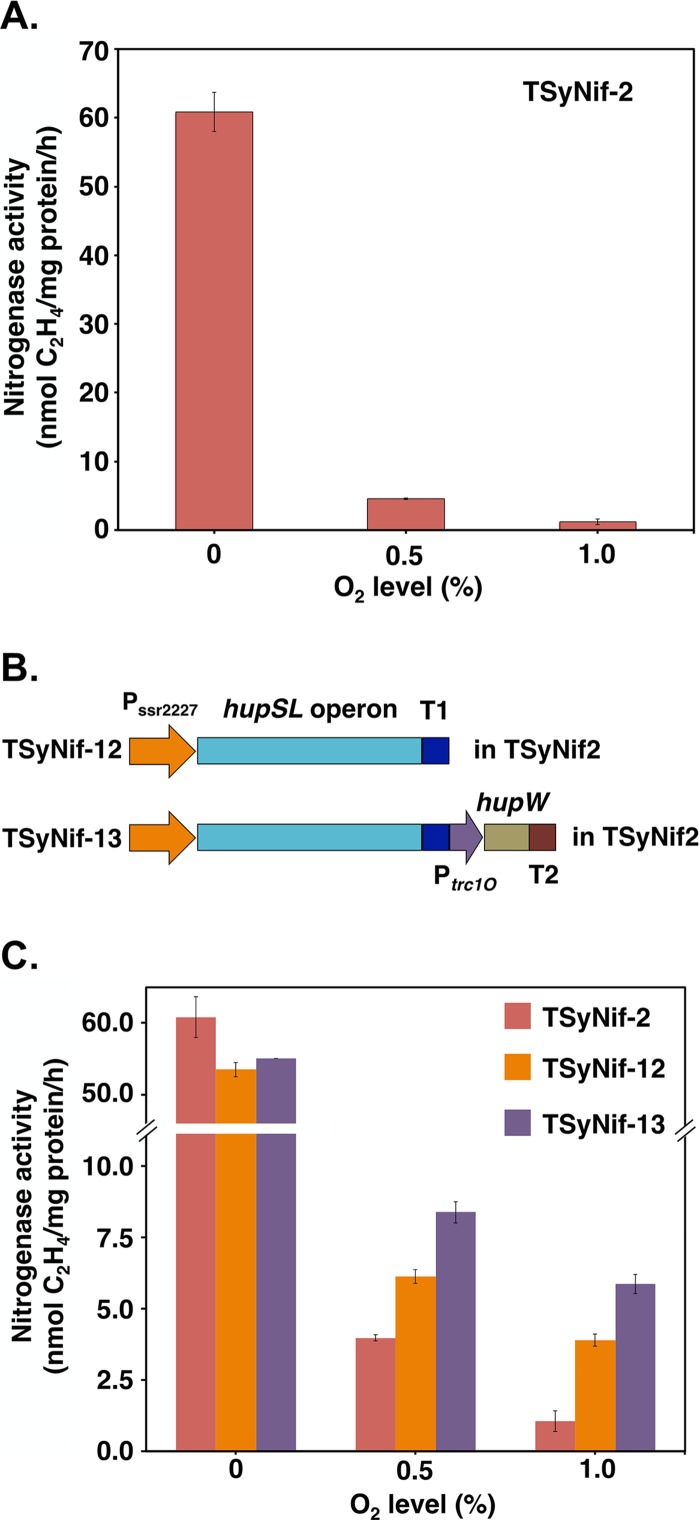
Expression of uptake hydrogenase improves O_2_ tolerance of nitrogenase. (A) Effect of O_2_ on nitrogen fixation activity of the TSyNif-2 strain. (B) Schematic showing the insertion of uptake hydrogenase genes *hupSL* and *hupW* from *Cyanothece* 51142 into the chromosome of the TSyNif-2 strain. (C) Comparison of nitrogen fixation activities under different micro-oxic conditions. Samples were collected from cultures under 12-h light/12-h dark conditions in BG11_0_ medium. Nitrogen fixation activity was assayed by acetylene reduction, and error bars represent the standard deviations of results from at least three independent experiments.

In order to test the oxygen sensitivity of nitrogen fixation activity in TSyNif-2, a measured amount of oxygen was added to the headspace of cultures grown in BG11_0_ media to generate micro-oxic conditions of 0.5% and 1.0% of O_2_ in the sealed testing bottles. The activity dropped more than 10-fold and 60-fold (Fig. 4A), respectively, demonstrating that, as expected, nitrogen fixation activity in engineered *Synechocystis* 6803 is highly sensitive to O_2_. To enhance O_2_ tolerance under the same conditions, genes coding for the uptake hydrogenase enzyme from *Cyanothece* 51142 were introduced into the chromosome of the TSyNif-2 strain. The uptake hydrogenase is conserved in diazotrophic cyanobacteria (38) and has been shown to be necessary for nitrogen fixation under aerobic conditions in *Cyanothece* (39). The uptake hydrogenase may utilize the H_2_ produced by the nitrogenase, but it may have three other beneficial functions for the organism: supplying the organism with ATP via the oxyhydrogen (Knallgas) reaction; removing oxygen from nitrogenase, thereby protecting it from inactivation; and providing electrons (reducing power) to nitrogenase and other enzymes (39). The structural genes for this hydrogenase, *hupS* and *hupL*, present together in a single operon in *Cyanothece* 51142, were transformed into TSyNif-2, generating strain TSyNif-12 (Fig. 4B). In addition to the structural genes *hupSL*, a protease encoded by *hupW* is also present in *Cyanothece* 51142. HupW is required for the maturation of HupL protein through the processing of its C terminus (40). Thus, the *hupSLW* genes organized in two operons were transformed into TSynif-2 to generate the TSyNif-13 strain (Fig. 4B). The expression of *hup* genes in TSyNif-12 and TSyNif-13 was assessed by RT-PCR (Fig. S6). The introduction of the uptake hydrogenase did not affect nitrogen fixation activity under anaerobic conditions (Fig. 4C). Interestingly, under micro-oxic conditions, nitrogen fixation activity markedly improved with the expression of uptake hydrogenase, especially for strain TSyNif-13, with 2-fold and 6-fold increases in TSyNif-2 for O_2_ levels of 0.5% and 1.0%, respectively. The results described above suggest that expression of uptake hydrogenase proves to be highly effective in enhancing O_2_ tolerance of nitrogen fixation activity in the engineered *Synechocystis* strain.

10.1128/mBio.01029-18.6 FIG S6  Transcriptional analysis of uptake hydrogenase genes. (A and B) RT-PCR analysis of *hupSL* and *hupW* genes in the (A) TSyNif-12 and (B) TSyNif-13 strains. Lanes 1 to 3 in both panels represent samples collected from BG11 medium, and lanes 4 to 6 represent samples collected from BG11_0_ medium. RNA was extracted from the cells grown under 12-h light/12-h dark conditions. M, molecular mass standards. Download FIG S6, PDF file, 1.3 MB. Copyright © 2018 Liu et al. 2018 Liu et al. This content is distributed under the terms of the Creative Commons Attribution 4.0 International license.

In this study, introduction of the *nif* gene cluster from *Cyanothece* 51142 enabled nitrogen fixation activity in *Synechocystis* 6803. The minimal cluster for 24 genes (Fig. 2) for nitrogenase activity will provide a useful framework for future studies to further enhance such activity by refactoring genes as previously done in Klebsiella oxytoca (34). Although uptake hydrogenase is a complex enzyme, introduction of its structural genes and a protease works as a mechanism providing protection from the toxicity of O_2_. The O_2_ toxicity to nitrogenase is likely the most difficult aspect to overcome to achieve nitrogen fixation activity under aerobic conditions.

A fully functional nitrogenase holoenzyme requires 8 electrons and 16 ATPs to reduce one molecule of N_2_ to ammonia. Thus, metabolism within cells needs to be adjusted to supply enough reducing power and energy for nitrogen fixation. Biosynthesis of fully functional nitrogenase is a complex process. This complexity increases the difficulty of finding the minimal genes and the best ratios of proteins expressed from the *nif* cluster in *Synechocystis* 6803. Genes with the same designations in different species occasionally have alternative functions (see Table S1 in the supplemental material). An example is the *nifX* gene, which functions as a negative regulator in Klebsiella oxytoca (35). Gene *nifX* is of importance in *Cyanothece*, since deletion of *nifX* affects nitrogenase activity.

10.1128/mBio.01029-18.7 TABLE S1  Impact of various *nif* gene inactivations on nitrogenase activity in different engineered strains. Relative levels of activities with respect to that determined for the respective engineered strain with wild-type *nif* cluster are indicated. Download TABLE S1, PDF file, 0.05 MB. Copyright © 2018 Liu et al. 2018 Liu et al. This content is distributed under the terms of the Creative Commons Attribution 4.0 International license.

Although multiple challenges and many barriers exist with respect to enabling plants to efficiently fix atmospheric nitrogen, we have engineered an oxygenic photosynthetic cell to fix N_2_ by reconfiguring the genetic processes for nitrogen fixation from *Cyanothece* 51142 to function in *Synechocystis* 6803. Our studies to date have established the highest rate of engineered nitrogen fixation activity in any nondiazotrophic oxygenic organism.

## MATERIALS AND METHODS

### Microorganisms, culture conditions, and media.

All cyanobacterial strains, including *Cyanothece* 51142, *Synechocystis* 6803, and engineered strains (see Table S2 in the supplemental material), were cultured in 100-ml flasks of fresh BG11 medium (41) with appropriate antibiotics (20 µg/ml kanamycin, 15 µg/ml chloramphenicol, or 20 µg/ml spectinomycin). As a preculture, cells were grown at 30°C, with 150 rpm shaking, and under conditions of 50 µmol photons ⋅ m^−2^ ⋅ s^−1^ constant light. For the nitrogen fixation activity assay, unless otherwise stated, precultured cells were collected and washed with fresh BG11_0_ medium (BG11 medium without nitrate) and resuspended in 500 ml fresh BG11_0_ medium. Cells were grown at 30°C with air bubbling under 12-h light/12-h dark conditions with 50 µmol photons ⋅ m^−2^ ⋅ s^−1^ of light. Yeast and E. coli strains (listed in Table S2) used for construction of recombinant plasmids were grown with 200 rpm shaking in yeast extract-peptone-dextrose plus adenine (YPAD) medium (42) and LB medium at 30°C and 37°C, respectively.

10.1128/mBio.01029-18.8 TABLE S2  Strains and plasmids used in this study. Download TABLE S2, PDF file, 0.1 MB. Copyright © 2018 Liu et al. 2018 Liu et al. This content is distributed under the terms of the Creative Commons Attribution 4.0 International license.

### Construction of recombinant plasmids and engineered strains.

Plasmids and strains used in this study are listed in Table S2, and all primers used in this study are listed in Table S3. Two methods were used to construct the plasmids: DNA assembler (32) and Gibson assembly (43). For building the large pSyNif-1 and pSyNif-2 plasmids containing the *nif* genes, genomic DNA from *Cyanothece* 51142 was used as the template for PCR, and all DNA fragments were combined using the DNA assembler method to form the plasmids in yeast. For the other recombinant plasmids listed in Table S2, Gibson assembly was the method used to construct them with DNA fragments amplified by PCR. Genomic DNAs from *Cyanothece* 51142 and the large pSyNif-2 plasmid were used as the templates for PCR to construct the plasmids for backbone replacement, the plasmids containing the uptake hydrogenase genes, or the plasmids used to remove specific *nif* genes. The sequences of all of the plasmids constructed in this study were verified (Genewiz, NJ).

10.1128/mBio.01029-18.9 TABLE S3  Primers used in this study. Download TABLE S3, PDF file, 0.1 MB. Copyright © 2018 Liu et al. 2018 Liu et al. This content is distributed under the terms of the Creative Commons Attribution 4.0 International license.

Plasmids pSyNif-1 and pSyNif-2 were introduced into the *Synechocystis* 6803 wild-type strain through the method of triparental conjugation (44) to form strains TSyNif-1 and TSyNif-2, respectively. The other recombinant plasmids were transformed into *Synechocystis* 6803 by natural transformation (45), and double homologous recombination integrated the fragments into the chromosome (for the uptake hydrogenase genes) or the plasmid (for the plasmid backbone replacement and for the specific removal of *nif* genes).

### Reverse transcription-PCR (RT-PCR) and quantitative PCR (q-PCR).

RT-PCR analysis was performed using RNA samples isolated from culture grown in BG11_0_ medium at time point D1 (1 h into the dark period) under light/dark conditions. After extraction and quantification of RNA (46), 100 ng of DNase-treated RNA samples and Superscript II reverse transcriptase and random primers (Invitrogen) were used for reverse transcription according to the manufacturer’s instructions. cDNA generated after reverse transcription was used as the template for PCR to validate the transcription of genes.

q-PCR was performed on RNA samples extracted from culture grown in BG11_0_ medium under light/dark conditions as previously described (27). Briefly, QRT-PCR Sybr green dUTP mix (ABgene) was used for the assay on an ABI 7500 system (Applied Biosystems). Each reaction was performed in three replicates, and the average threshold cycle (*C*_*T*_) value was used to calculate the relative transcriptional levels for the amounts of RNA. All primers used for RT-PCR and q-PCR are listed in Table S3.

### Measurement of nitrogen fixation activity.

Nitrogen fixation activity was measured by an acetylene reduction assay (47) modified from a previously published method (48). Unless otherwise stated, the activity assay was performed as follows. A 25-ml volume of cyanobacterial culture was grown in BG11_0_ medium with air bubbling under light/dark conditions as mentioned above and was transferred to a 125-ml air-tight glass vial. DCMU (10 μM) was added to the culture, vials were flushed with pure argon, and cultures were incubated under 12-h light/12-h dark conditions. Cells in the sealed vials were cultured overnight, and, at the D1 time point, 5 ml acetylene was added into the sealed vials, followed by 3 h of incubation in light at 30°C. Two hundred microliters of gas was sampled from the headspace and injected into an Agilent 6890 N gas chromatograph equipped with a Porapak N column and a flame ionization detector, using argon as the carrier gas. The temperatures of the detector, injector, and oven were 200°C, 150°C, and 100°C, respectively.

Total protein levels were determined on a plate reader (Bio-Tek Instruments, Winooski, VT) using a bicinchoninic acid (BCA) assay kit (Pierce, Rockford, IL) according to the manufacturer’s instructions. Total chlorophyll *a* was subjected to methanol extraction and quantification on an Olis DW2000 spectrophotometer (OnLine Instrument Systems, Inc., GA).

### *In vivo* ^15^N_2_ incorporation assay.

All strains were grown under light/dark conditions as mentioned above, and 50-ml cultures were transferred into a 125-ml airtight glass vial. DCMU (10 μM) was added to the culture, vials were flushed with pure N_2_, and cultures were incubated under light/dark conditions. Cells in the sealed vials were cultured overnight, and, at the D1 time point, 8 ml of headspace gas was removed followed by injection of 8 ml of ^15^N_2_ gas (Cambridge Isotope Laboratories, Inc.) (98%^+^). After 8 h of incubation at 30°C in light (50 µmol photons ⋅ m^−2^ ⋅ s^−1^), the cultures were collected and dried in a laboratory oven at 50°C to 60°C for 24 h. The dried pellets were ground, weighed, and sealed into tin capsules. Isotope ratios were measured by elemental analyzer-isotope ratio mass spectrometry (EA-IRMS; Thermo Fisher Scientific), and values are indicated as δ^15^N (‰), where the number represents a linear transform of the ^15^N/^14^N isotope ratios, representing the per-mille difference between the isotope ratios in a sample and in atmospheric N_2_ (49). Data presented are mean values determined on the basis of results from at least two biological replicate cultures.

### Western blot analysis.

The *nifH* (cce_0559) gene and *nifD* (cce_0560) gene were subjected to individual PCR amplifications from the genomic DNA of *Cyanothece* 51142, using the primers shown in Table S3. The PCR fragment was ligated into expression vector pET28a cleaved by NdeI and BamHI. The resulting plasmids (pET28a-nifH and pET28a-nifD) were used to produce NifH and NifD proteins, each with an N-terminal His_6_ tag. For overproduction of these proteins, E. coli BL21 (DE3) was transformed with plasmids pET28a-nifH and pET28a-nifD, respectively, and cultivated in LB medium at 37°C to an optical density at 600 nm (*A*_600_) of 0.3. Protein expression was induced by the addition of 0.2 mM isopropyl-β*-*d-thiogalactopyranoside, and the culture was incubated for another 18 h at 20°C. After the cells were harvested, NifH and NifD were individually purified by nickel-nitrilotriacetic acid (Ni-NTA) affinity chromatography. Briefly, harvested E. coli cells were resuspended in 20 mM HEPES buffer (pH 7.0) containing 100 mM NaCl and in 2 mM β-mercaptoethanol supplemented with a protease inhibitor cocktail (Sigma-Aldrich). Lysozyme was added to reach a concentration of 1 mg/ml, and the cells were lysed by freezing-thawing, followed by sonication. After cells were centrifuged at 13,000 rpm, the Tris-HCl buffer (pH 8.0) was added to the supernatant (final concentration, 50 mM), and it was loaded onto a Ni-NTA agarose column (0.2 ml). After bound proteins were washed with the starting buffer containing 1 M NaCl, they were eluted with 0.3 ml of the starting buffer containing 250 mM imidazole. The purified protein was stored at −20°C and used as the positive control for Western blot assay.

Cyanobacterial cells cultured in N-free medium under conditions of light/dark cycles were collected at time point D4 (4 h after the dark phase) and resuspended in 0.5 ml TG buffer (10 mM Tris-HCl [pH 8.0], 10% glycerol) containing a protease inhibitor cocktail (Sigma-Aldrich). A 0.5-ml volume of sterilized, acid-washed glass beads was added to the cells, and the mixture was disrupted using a bead beater (BioSpec Products). The resultant mixture was centrifuged for 10 min at 7,500 × *g*, and the supernatant was transferred into a new tube. The amount of protein was determined using bicinchoninic acid (BCA) protein assay reagent (Thermo Scientific).

A 15-µg (total) protein extract from each sample was solubilized with 8× sample buffer (10 ml of 0.5 M Tris [pH 6.8], 15 ml of 70% glycerol, 8 ml of 20% sodium dodecyl sulfate, 4 ml of β-mercaptoethanol, 4 ml of 0.1% bromophenol blue) at 70°C for 10 min and separated on a sodium dodecyl sulfate (0.1% [wt/vol])-polyacrylamide (12.5% [wt/vol]) gel by electrophoresis. After electrophoresis, proteins were transferred to a polyvinylidene difluoride (PVDF) membrane (Millipore), blocked using 5% bovine serum albumin (BSA) for 2 h at room temperature, and then separately incubated with the primary rabbit antibodies raised against NifH and NifD protein of Rhodospirillum rubrum (50, 51) diluted in 1.5% BSA (1:2,000) overnight at 4°C. The horseradish peroxidase (HRP)-conjugated secondary antibody goat anti-rabbit IgG (H+L)-HRP conjugate (Bio-Rad) was diluted at 1:5,000 in 1.5% BSA. Immunodetection was performed using Western blotting Luminol reagent (Millipore).

## References

[1] Stokstad E 2016 The nitrogen fix. Science 353:1225–1227. doi:10.1126/science.353.6305.1225. 27634521

[2] Good A 2018 Toward nitrogen-fixing plants. Science 359:869–870. doi:10.1126/science.aas8737. 29472469

[3] Vicente EJ, Dean DR 2017 Keeping the nitrogen-fixation dream alive. Proc Natl Acad Sci U S A 114:3009–3011. doi:10.1073/pnas.1701560114. 28283657 PMC5373348

[4] Burén S, Rubio LM 2018 State of the art in eukaryotic nitrogenase engineering. FEMS Microbiol Lett 365:fnx274. doi:10.1093/femsle/fnx274. PMC5812491 29240940

[5] Curatti L, Rubio LM 2014 Challenges to develop nitrogen-fixing cereals by direct *nif*-gene transfer. Plant Sci 225:130–137. doi:10.1016/j.plantsci.2014.06.003. 25017168

[6] Dos Santos PC, Fang Z, Mason SW, Setubal JC, Dixon R 2012 Distribution of nitrogen fixation and nitrogenase-like sequences amongst microbial genomes. BMC Genomics 13:162. doi:10.1186/1471-2164-13-162. 22554235 PMC3464626

[7] Hu Y, Ribbe MW 2015 Nitrogenase and homologs. J Biol Inorg Chem 20:435–445. doi:10.1007/s00775-014-1225-3. 25491285 PMC4336585

[8] Mus F, Alleman AB, Pence N, Seefeldt LC, Peters JW 2018 Exploring the alternatives of biological nitrogen fixation. Metallomics 10:523–538. doi:10.1039/c8mt00038g. 29629463

[9] Hu Y, Ribbe MW 2013 Nitrogenase assembly. Biochim Biophys Acta 1827:1112–1122. doi:10.1016/j.bbabio.2012.12.001. 23232096 PMC3622157

[10] Sickerman NS, Ribbe MW, Hu Y 2017 Nitrogenase cofactor assembly: an elemental inventory. Acc Chem Res 50:2834–2841. doi:10.1021/acs.accounts.7b00417. 29064664

[11] Sickerman NS, Rettberg LA, Lee CC, Hu Y, Ribbe MW 2017 Cluster assembly in nitrogenase. Essays Biochem 61:271–279. doi:10.1042/EBC20160071. 28487403

[12] Sickerman NS, Hu Y, Ribbe MW 2017 Nitrogenase assembly: strategies and procedures. Methods Enzymol 595:261–302. doi:10.1016/bs.mie.2017.07.006. 28882203

[13] Dixon RA, Postgate JR 1972 Genetic transfer of nitrogen fixation from *Klebsiella pneumoniae* to *Escherichia coli*. Nature 237:102–103. doi:10.1038/237102a0. 4555442

[14] Wang L, Zhang L, Liu Z, Zhao D, Liu X, Zhang B, Xie J, Hong Y, Li P, Chen S, Dixon R, Li J 2013 A minimal nitrogen fixation gene cluster from *Paenibacillus* sp. WLY78 enables expression of active nitrogenase in *Escherichia coli*. PLoS Genet 9:e1003865. doi:10.1371/journal.pgen.1003865. 24146630 PMC3798268

[15] Yang J, Xie X, Wang X, Dixon R, Wang YP 2014 Reconstruction and minimal gene requirements for the alternative iron-only nitrogenase in *Escherichia coli*. Proc Natl Acad Sci U S A 111:E3718–E3725. doi:10.1073/pnas.1411185111. 25139995 PMC4156695

[16] Smanski MJ, Bhatia S, Zhao D, Park Y, Woodruff LBA, Giannoukos G, Ciulla D, Busby M, Calderon J, Nicol R, Gordon DB, Densmore D, Voigt CA 2014 Functional optimization of gene clusters by combinatorial design and assembly. Nat Biotechnol 32:1241–1249. doi:10.1038/nbt.3063. 25419741

[17] Yang J, Xie X, Yang M, Dixon R, Wang YP 2017 Modular electron-transport chains from eukaryotic organelles function to support nitrogenase activity. Proc Natl Acad Sci U S A 114:E2460–E2465. doi:10.1073/pnas.1620058114. 28193863 PMC5373397

[18] Cheng Q, Day A, Dowson-Day M, Shen GF, Dixon R 2005 The *Klebsiella pneumoniae* nitrogenase Fe protein gene (*nifH*) functionally substitutes for the *chlL* gene in *Chlamydomonas reinhardtii*. Biochem Biophys Res Commun 329:966–975. doi:10.1016/j.bbrc.2005.02.064. 15752750

[19] Zamir A, Maina CV, Fink GR, Szalay AA 1981 Stable chromosomal integration of the entire nitrogen fixation gene cluster from *Klebsiella pneumoniae* in yeast. Proc Natl Acad Sci U S A 78:3496–3500. doi:10.1073/pnas.78.6.3496. 6267596 PMC319596

[20] López-Torrejón G, Jiménez-Vicente E, Buesa JM, Hernandez JA, Verma HK, Rubio LM 2016 Expression of a functional oxygen-labile nitrogenase component in the mitochondrial matrix of aerobically grown yeast. Nat Commun 7:11426. doi:10.1038/ncomms11426. 27126134 PMC4855529

[21] Pérez-González A, Kniewel R, Veldhuizen M, Verma HK, Navarro-Rodríguez M, Rubio LM, Caro E 2017 Adaptation of the GoldenBraid modular cloning system and creation of a toolkit for the expression of heterologous proteins in yeast mitochondria. BMC Biotechnol 17:80. doi:10.1186/s12896-017-0393-y. 29132331 PMC5683533

[22] Burén S, Young EM, Sweeny EA, Lopez-Torrejón G, Veldhuizen M, Voigt CA, Rubio LM 2017 Formation of nitrogenase NifDK tetramers in the mitochondria of *Saccharomyces cerevisiae*. Synth Biol 6:1043–1055. doi:10.1021/acssynbio.6b00371. PMC5477005 28221768

[23] Allen RS, Tilbrook K, Warden AC, Campbell PC, Rolland V, Singh SP, Wood CC 2017 Expression of 16 nitrogenase proteins within the plant mitochondrial matrix. Front Plant Sci 8:287. doi:10.3389/fpls.2017.00287. 28316608 PMC5334340

[24] Ivleva NB, Groat J, Staub JM, Stephens M 2016 Expression of active subunit of nitrogenase via integration into plant organelle genome. PLoS One 11:e0160951. doi:10.1371/journal.pone.0160951. 27529475 PMC4986947

[25] Oldroyd GE, Dixon R 2014 Biotechnological solutions to the nitrogen problem. Curr Opin Biotechnol 26:19–24. doi:10.1016/j.copbio.2013.08.006. 24679253

[26] Falcón LI, Magallón S, Castillo A 2010 Dating the cyanobacterial ancestor of the chloroplast. ISME J 4:777–783. doi:10.1038/ismej.2010.2. 20200567

[27] Stöckel J, Welsh EA, Liberton M, Kunnvakkam R, Aurora R, Pakrasi HB 2008 Global transcriptomic analysis of *Cyanothece* 51142 reveals robust diurnal oscillation of central metabolic processes. Proc Natl Acad Sci U S A 105:6156–6161. doi:10.1073/pnas.0711068105. 18427117 PMC2329701

[28] Červený J, Sinetova MA, Valledor L, Sherman LA, Nedbal L 2013 Ultradian metabolic rhythm in the diazotrophic cyanobacterium *Cyanothece* sp. ATCC 51142. Proc Natl Acad Sci U S A 110:13210–13215. doi:10.1073/pnas.1301171110. 23878254 PMC3740830

[29] Bandyopadhyay A, Elvitigala T, Welsh E, Stöckel J, Liberton M, Min H, Sherman LA, Pakrasi HB 2011 Novel metabolic attributes of the genus *Cyanothece*, comprising a group of unicellular nitrogen-fixing cyanothece. MBio 2:e00214. doi:10.1128/mBio.00214-11. 21972240 PMC3187577

[30] Bandyopadhyay A, Elvitigala T, Liberton M, Pakrasi HB 2013 Variations in the rhythms of respiration and nitrogen fixation in members of the unicellular diazotrophic cyanobacterial genus *Cyanothece*. Plant Physiol 161:1334–1346. doi:10.1104/pp.112.208231. 23274238 PMC3585600

[31] Shih PM, Wu D, Latifi A, Axen SD, Fewer DP, Talla E, Calteau A, Cai F, Tandeau de Marsac N, Rippka R, Herdman M, Sivonen K, Coursin T, Laurent T, Goodwin L, Nolan M, Davenport KW, Han CS, Rubin EM, Eisen JA, Woyke T, Gugger M, Kerfeld CA 2013 Improving the coverage of the cyanobacterial phylum using diversity-driven genome sequencing. Proc Natl Acad Sci U S A 110:1053–1058. doi:10.1073/pnas.1217107110. 23277585 PMC3549136

[32] Shao Z, Zhao H, Zhao H 2009 DNA assembler, an *in vivo* genetic method for rapid construction of biochemical pathways. Nucleic Acids Res 37:e16. doi:10.1093/nar/gkn991. 19074487 PMC2632897

[33] Taton A, Unglaub F, Wright NE, Zeng WY, Paz-Yepes J, Brahamsha B, Palenik B, Peterson TC, Haerizadeh F, Golden SS, Golden JW 2014 Broad-host-range vector system for synthetic biology and biotechnology in cyanobacteria. Nucleic Acids Res 42:e136. doi:10.1093/nar/gku673. 25074377 PMC4176158

[34] Temme K, Zhao D, Voigt CA 2012 Refactoring the nitrogen fixation gene cluster from *Klebsiella oxytoca*. Proc Natl Acad Sci U S A 109:7085–7090. doi:10.1073/pnas.1120788109. 22509035 PMC3345007

[35] Gosink MM, Franklin NM, Roberts GP 1990 The product of the *Klebsiella pneumoniae nifX* gene is a negative regulator of the nitrogen fixation (*nif*) regulon. J Bacteriol 172:1441–1447. doi:10.1128/jb.172.3.1441-1447.1990. 2155202 PMC208618

[36] Ng AH, Berla BM, Pakrasi HB 2015 Fine-tuning of photoautotrophic protein production by combining promoters and neutral sites in the cyanobacterium *Synechocystis* sp. strain PCC 6803. Appl Environ Microbiol 81:6857–6863. doi:10.1128/AEM.01349-15. 26209663 PMC4561709

[37] Liu D, Pakrasi HB 2018 Exploring native genetic elements as plug-in tools for synthetic biology in the cyanobacterium *Synechocystis* sp. PCC 6803. Microb Cell Fact 17:48. doi:10.1186/s12934-018-0897-8. 29580240 PMC5868059

[38] Tamagnini P, Leitão E, Oliveira P, Ferreira D, Pinto F, Harris DJ, Heidorn T, Lindblad P 2007 Cyanobacterial hydrogenases: diversity, regulation and applications. FEMS Microbiol Rev 31:692–720. doi:10.1111/j.1574-6976.2007.00085.x. 17903205

[39] Zhang X, Sherman DM, Sherman LA 2014 The uptake hydrogenase in the unicellular diazotrophic cyanobacterium *Cyanothece* sp. strain PCC 7822 protects nitrogenase from oxygen toxicity. J Bacteriol 196:840–849. doi:10.1128/JB.01248-13. 24317398 PMC3911175

[40] Wünschiers R, Batur M, Lindblad P 2003 Presence and expression of hydrogenase specific C-terminal endopeptidases in cyanobacteria. BMC Microbiol 3:8. doi:10.1186/1471-2180-3-8. 12735794 PMC156652

[41] Stanier RY, Deruelles J, Rippka R, Herdman M, Waterbury JB 1979 Generic assignments, strain histories and properties of pure cultures of cyanobacteria. Microbiology 111:1–61. doi:10.1099/00221287-111-1-1.

[42] Bergkessel M, Guthrie C 2013 Chemical transformation of yeast. Methods Enzymol 529:311–320. doi:10.1016/B978-0-12-418687-3.00026-4. 24011057

[43] Gibson DG, Young L, Chuang RY, Venter JC, Hutchison CA III, Smith HO 2009 Enzymatic assembly of DNA molecules up to several hundred kilobases. Nat Methods 6:343–345. doi:10.1038/nmeth.1318. 19363495

[44] Golden SS, Brusslan J, Haselkorn R 1987 Genetic engineering of the cyanobacterial chromosome. Methods Enzymol 153:215–231. 3123881 10.1016/0076-6879(87)53055-5

[45] Williams JGK 1988 Construction of specific mutations in photosystem II photosynthetic reaction center by genetic engineering methods in *Synechocystis* 6803. Methods Enzymol 167:766–778.

[46] Kruse O, Rupprecht J, Mussgnug JH, Dismukes GC, Hankamer B 2005 Photosynthesis: a blueprint for solar energy capture and biohydrogen production technologies. Photochem Photobiol Sci 4:957–970. doi:10.1039/b506923h. 16307108

[47] Oda Y, Samanta SK, Rey FE, Wu L, Liu X, Yan T, Zhou J, Harwood CS 2005 Functional genomic analysis of three nitrogenase isozymes in the photosynthetic bacterium *Rhodopseudomonas palustris*. J Bacteriol 187:7784–7794. doi:10.1128/JB.187.22.7784-7794.2005. 16267302 PMC1280311

[48] Bandyopadhyay A, Stöckel J, Min H, Sherman LA, Pakrasi HB 2010 High rates of photobiological H_2_ production by a cyanobacterium under aerobic conditions. Nat Commun 1:139. doi:10.1038/ncomms1139. 21266989

[49] Montoya JP, Voss M, Kahler P, Capone DG 1996 A simple, high-precision, high-sensitivity tracer assay for N_2_ fixation. Appl Environ Microbiol 62:986–993. 16535283 10.1128/aem.62.3.986-993.1996 PMC1388808

[50] Colón-López MS, Sherman DM, Sherman LA 1997 Transcriptional and translational regulation of nitrogenase in light-dark- and continuous-light-grown cultures of the unicellular cyanobacterium *Cyanothece* sp. strain ATCC 51142. J Bacteriol 179:4319–4327. doi:10.1128/jb.179.13.4319-4327.1997. 9209050 PMC179256

[51] Grunwald SK, Lies DP, Roberts GP, Ludden PW 1995 Posttranslational regulation of nitrogenase in *Rhodospirillum rubrum* strains overexpressing the regulatory enzymes dinitrogenase reductase ADP-ribosyltransferase and dinitrogenase reductase activating glycohydrolase. J Bacteriol 177:628–635. doi:10.1128/jb.177.3.628-635.1995. 7836296 PMC176637

[52] Curatti L, Brown CS, Ludden PW, Rubio LM 2005 Genes required for rapid expression of nitrogenase activity in *Azotobacter vinelandii*. Proc Natl Acad Sci U S A 102:6291–6296. doi:10.1073/pnas.0501216102. 15845763 PMC1088376

[53] Han Y, Lu N, Chen Q, Zhan Y, Liu W, Lu W, Zhu B, Lin M, Yang Z, Yan Y 2015 Interspecies transfer and regulation of Pseudomonas stutzeri A1501 nitrogen fixation island in *Escherichia coli*. J Microbiol Biotechnol 25:1339–1348. doi:10.4014/jmb.1502.02027. 25824431

[54] Kavanagh EP, Hill S 1993 Oxygen inhibition of nitrogenase activity in *Klebsiella pneumoniae*. J Gen Microbiol 139:1307–1314. doi:10.1099/00221287-139-6-1307. 8360623

