# Supplementary figures and images for: Engineering Nitrogen Fixation Activity in an Oxygenic Phototroph
Source: mBio. 2018 Jun 5;9(3):e01029-18. doi: 10.1128/mBio.01029-18 (PMC5989072; doi:10.1128/mBio.01029-18)

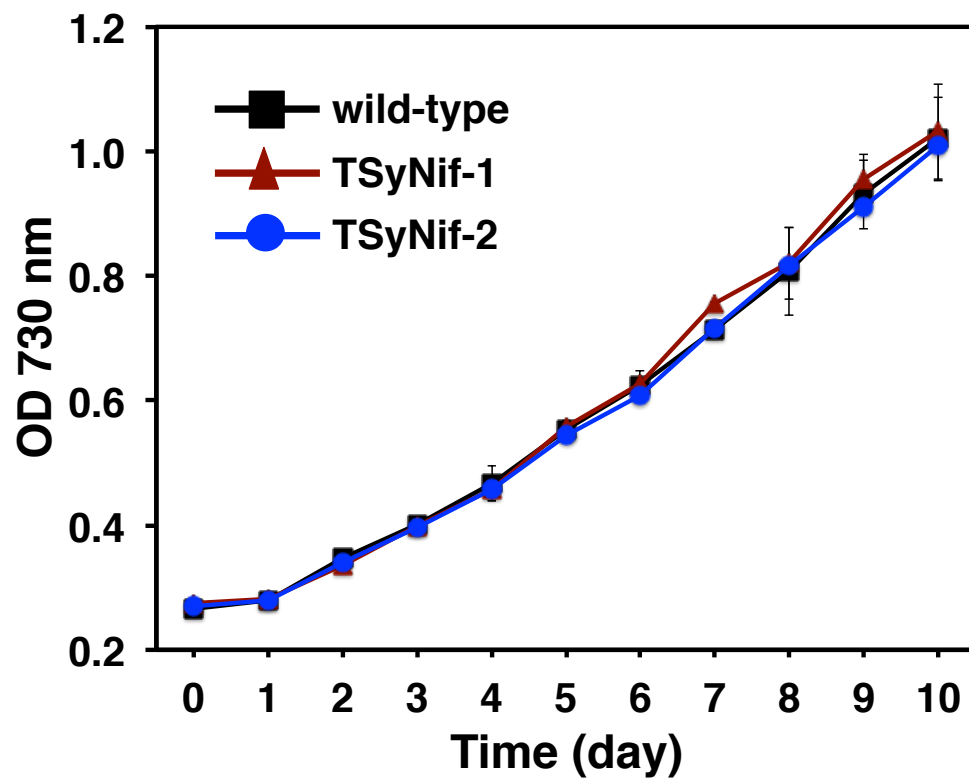

Supplement: FIG S1 [file mbo003183927sf1.pdf]

**A.**

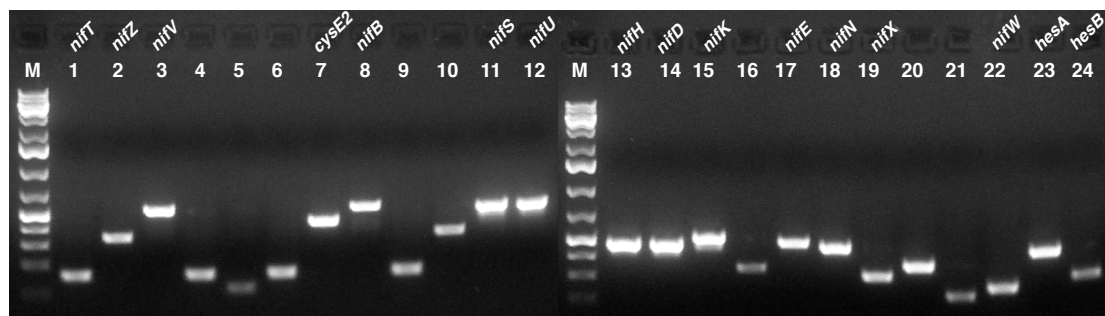

**B.**

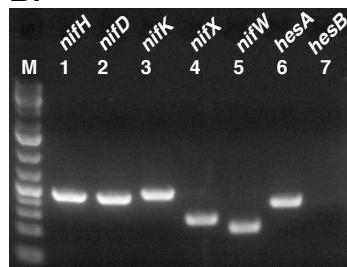

**C.**

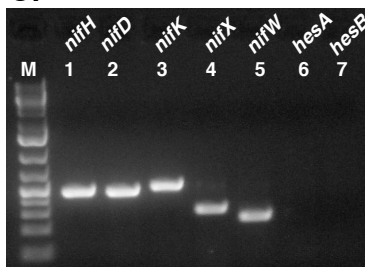

**D.**

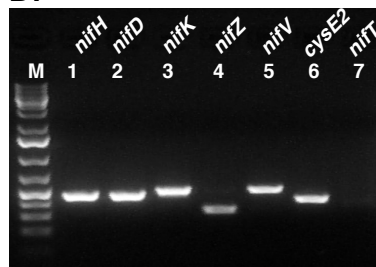

**E.**

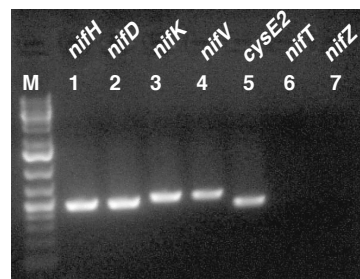

**F.**

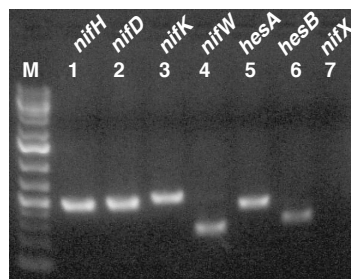

**G.**

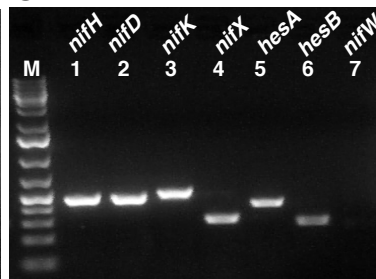

Supplement: FIG S3 [file mbo003183927sf3.pdf]

A.

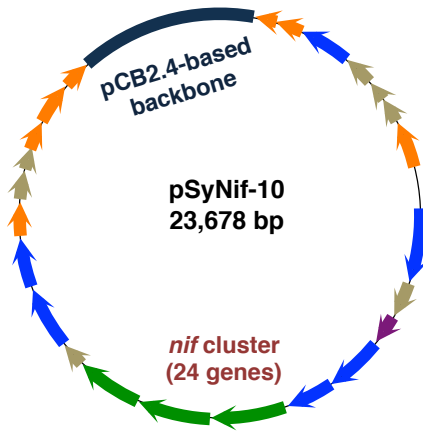

B.

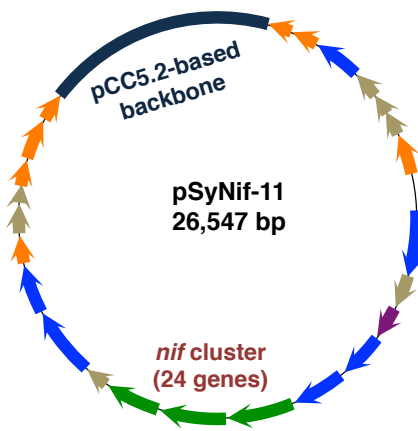

C.

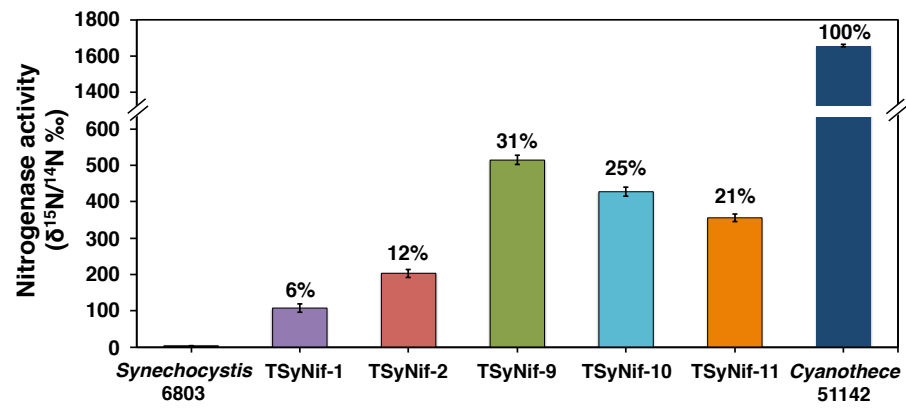

D.

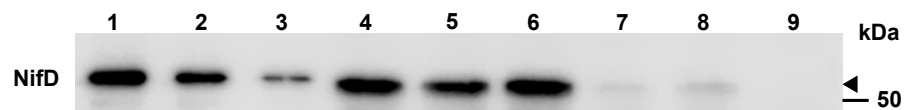

Supplement: FIG S4 [file mbo003183927sf4.pdf]

**A.**

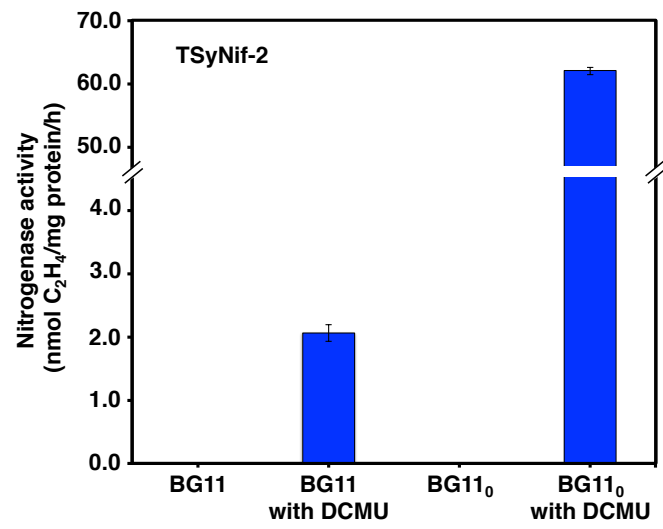

**B.**

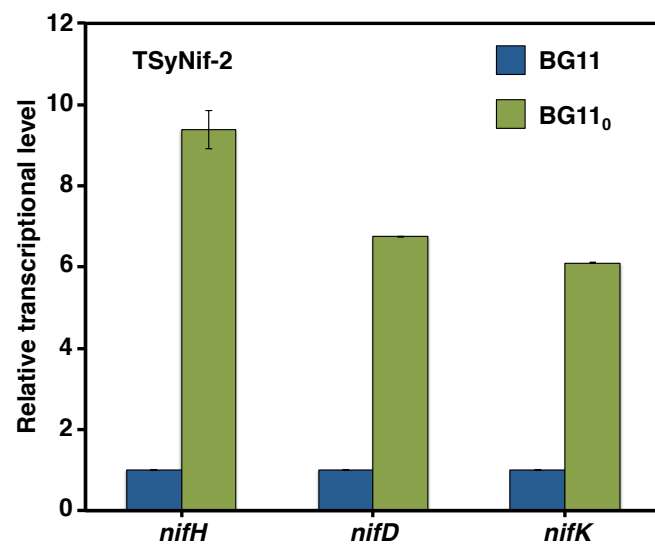

Supplement: FIG S5 [file mbo003183927sf5.pdf]

**A.**

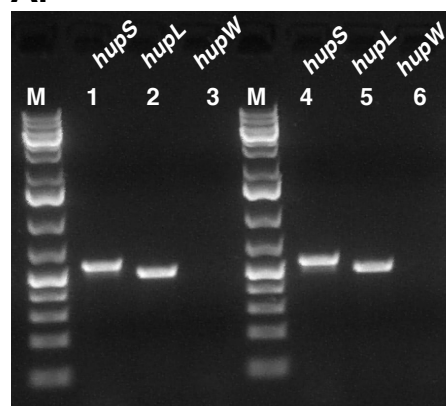

**B.**

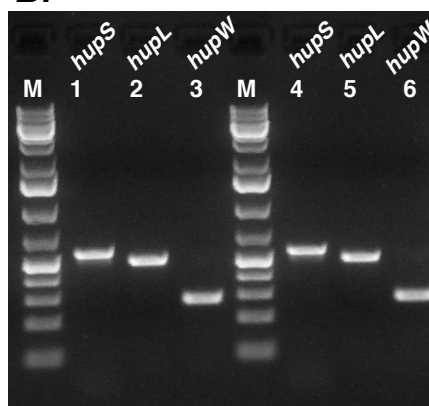

Supplement: FIG S6 [file mbo003183927sf6.pdf]
