# Supplementary material for: Engineering Nitrogen Fixation Activity in an Oxygenic Phototroph
Source: mBio. 2018 Jun 5;9(3):e01029-18. doi: 10.1128/mBio.01029-18 (PMC5989072; doi:10.1128/mBio.01029-18)
Supplement: FIG S2 [file mbo003183927sf2.pdf]

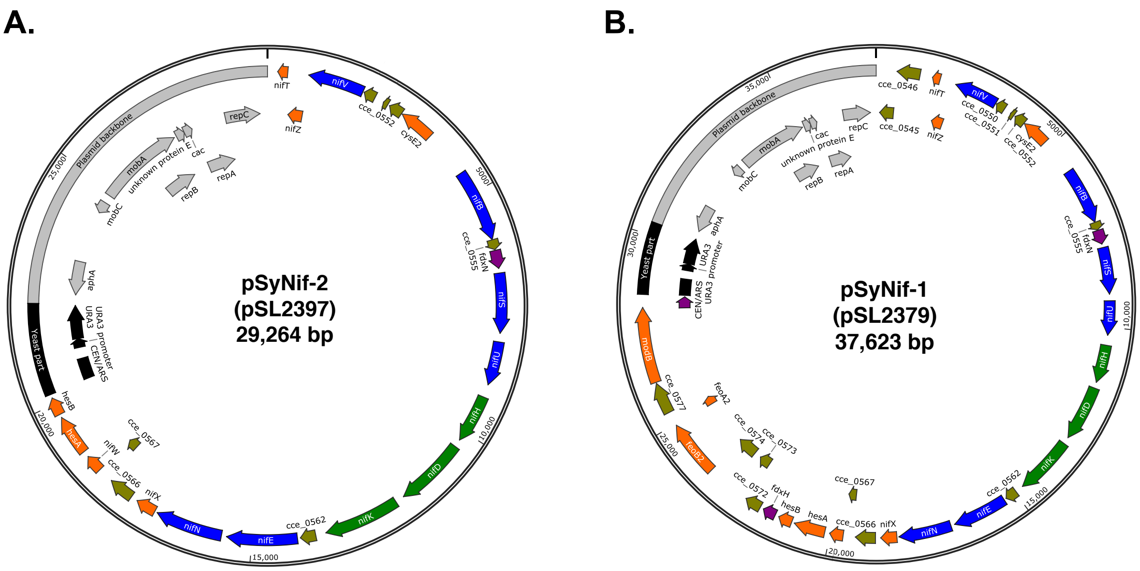
A.
pSyNif-2
(pSL2397)
29,264 bp
B.
pSyNif-1
(pSL2379)
37,623 bp
