# Supplementary material for: Engineering Nitrogen Fixation Activity in an Oxygenic Phototroph
Source: mBio. 2018 Jun 5;9(3):e01029-18. doi: 10.1128/mBio.01029-18 (PMC5989072; doi:10.1128/mBio.01029-18)
Supplement: TABLE S2 [file mbo003183927st2.pdf]

Table S2 Strains and plasmids used in this study

Strains and plasmids	Description	Source
**Strain**		
Saccharomyces. cerevisiae	Host for DNA assembly and integration	(3)
E. coli strain WM1788	Used for DNA cloning	(4)
E. coli strain Top 10	Used for DNA cloning	Our lab
Cyanothece sp. ATCC 51142	wild-type strain	Our lab
Synechocystis sp. PCC 6803	wild-type strain	Our lab
TSyNif-1 (T2379)	Containing entire *nif*-cluster 35 genes from *Cyanothece*	This study
TSyNif-2 (T2397)	Containing 24 genes of *nif*-cluster from *Cyanothece*	This study
TSyNif-3 (T2452)	T2397 without gene *hesB*	This study
TSyNif-4 (T2453)	T2397 without genes *hesAB*	This study
TSyNif-5 (T2454)	T2397 without gene *nifT*	This study
TSyNif-6 (T2466)	T2397 without gene *niTZ*	This study
TSyNif-7 (T2467)	T2397 without gene *nifX*	This study
TSyNif-8 (T2468)	T2397 without genes *nifW*	This study
TSyNif-9 (T2872)	Containing 24 genes of *nif*-cluster on pCA2.4 plasmid	This study
TSyNif-10 (T2873)	Containing 24 genes of *nif*-cluster on pCB2.4 plasmid	This study
TSyNif-11 (T2874)	Containing 24 genes of *nif*-cluster on pCC5.2 plasmid	This study
TSyNif-12 (T2444)	Genes *hupSL* from *Cyanothece* 51142 under $P_{ssr2227}$ in	This study
TSyNif-13 (T2537)	Gene *hupW* from *Cyanothece* under P_{trc1O} in T2444	This study
**Plasmid**		
pRL443	Used for conjugation	(5)
pRL663	Used for conjugation	(6)
pRSF1010	Broad-host-range shuttle vector	(7)
pUC118	DNA cloning vector	(8)
pSyNif-1 (pSL2379)	Containing entire *nif*-cluster 35 genes from *Cyanothece*	This study
pSyNif-2 (pSL2397)	Containing 24 genes of *nif*-cluster from *Cyanothece*	This study
pSyNif-3 (pSL2452)	Knocking-out plasmid for gene *hesB*	This study
pSyNif-4 (pSL2453)	Knocking-out plasmid for genes *hesAB*	This study
pSyNif-5 (pSL2454)	Knocking-out plasmid for gene *nifT*	This study
pSyNif-6 (pSL2466)	Knocking-out plasmid for gene *nifTZ*	This study
pSyNif-7 (pSL2467)	Knocking-out plasmid for gene *nifX*	This study
pSyNif-8 (pSL2468)	Knocking-out plasmid for genes *nifW*	This study
pSyNif-9 (pSL2872)	Containing 24 genes of *nif*-cluster on pCA2.4 plasmid	This study
pSyNif-10 (pSL2873)	Containing 24 genes of *nif*-cluster on pCB2.4 plasmid	This study
pSyNif-11 (pSL2874)	Containing 24 genes of *nif*-cluster on pCC5.2 plasmid	This study
pSyNif-12 (pSL2444)	Genes *hupSL* from *Cyanothece* 51142 under $P_{ssr2227}$	This study
pSyNif-13 (pSL2537)	Gene *hupW* from *Cyanothece* 51142 under P_{trc1O}	This study

REFERENCES

1. Yang J, Xie X, Wang X, Dixon R, Wang Y. 2014. Reconstruction and minimal gene requirements for the alternative iron-only nitrogenase in *Escherichia coli*. Proc Natl Acad Sci U S A 111:E3718-3725.

2. Wang L, Zhang L, Liu Z, Zhao D, Liu X, Zhang B, Xie J, Hong Y, Li P, Chen S, Dixon R, Li J. 2013. A minimal nitrogen fixation gene cluster from *Paenibacillus* sp. WLY78 enables expression of active nitrogenase in *Escherichia coli*. PLoS Genet 9:e1003865.

3. Shao Z, Zhao H, Zhao H. 2009. DNA assembler, an *in vivo* genetic method for rapid construction of biochemical pathways. Nucleic Acids Res. 37:e16.

4. Datsenko KA, Wanner BL. 2000. One-step inactivation of chromosomal genes in *Escherichia coli* K-12 using PCR products. Proc Natl Acad Sci U S A 97:6640-6645.

5. Elhai J, Vepritskiy A, Muro-Pastor AM, Flores E, Wolk CP. 1997. Reduction of conjugal transfer efficiency by three restriction activities of *Anabaena sp.* strain PCC 7120. J Bacteriol 179:1998-2005.

6. Wang D, Meier TI, Chan CL, Feng G, Lee DN, Landick R. 1995. Discontinuous movements of DNA and RNA in RNA polymerase accompany formation of a paused transcription complex. Cell 81:341-350.

7. Huang HH, Camsund D, Lindblad P, Heidorn T. 2010. Design and characterization of molecular tools for a synthetic biology approach towards developing cyanobacterial biotechnology. Nucleic Acids Res 38:2577-2593.

8. Vieira J, Messing J. 1987. Production of single-stranded plasmid DNA. Methods Enzymol 153:3-11.
