# Supplementary material for: Engineering Nitrogen Fixation Activity in an Oxygenic Phototroph
Source: mBio. 2018 Jun 5;9(3):e01029-18. doi: 10.1128/mBio.01029-18 (PMC5989072; doi:10.1128/mBio.01029-18)
Supplement: TABLE S3 [file mbo003183927st3.pdf]

Table S3 Primers used in this study

Primer	Sequence (5′-3′)
SS001FRAG1_F	GCAAGACGTTTCCCGTTGAATATGGCTCATAATGCTGCTTCTTCGATGAT
SS001FRAG1_R	ACAGAAATACAAACCCCTCACCTG
SS001FRAG2_F	TGTAAACCTGGATAGCAACACACC
SS001FRAG2_R	TAGAACCCTCGAACGCTACGTC
SS001FRAG3_F	AAGTCACCCTACAAGGAGCCTG
SS001FRAG3_R	ACGGAGCATGGTGTGTCCTAATAC
SS001FRAG4_F	TCCAAGGAAATCTGAATACGAGGG
SS001FRAG4_R	ACAGCGTCCTTTCTTTGAAGTC
SS001FRAG5_F	ATGAACTTCACCCCTACTGAACTC
SS001FRAG5_R	ATGAGTAAACTGCTGGTTCCGG
SS001FRAG6_F	AGTGGTTCCTCTCAACTTAGCC
SS001FRAG6_R	CGTGATGAAAAGGACCCAGGTGGCACTTTTCGCCCAACTTAACCGAAGAG
SS002FRAGYT_F	CGAAAAGTGCCACCTGGGTC
SS002FRAGYT_R	GTGAGTTTAGTATACATGCA
SS002FRAGBK_F	AGTAAATGCATGTATACTAAACTCACCTGCAGCAATGGCAACAACG
SS002FRAGBK_R	TTAATCATGACATCATCATCGAAGAAGCAGCATTATGAGCCATATTCAAC
SS002FRAG1_F	GGCCGCTTTCCTGGCTTTGCTTCCCAATTAAAATGACAATTAAGTTCC
SS002FRAG1_R	GACTGTAAGAGGAATTGTCAGAGGC
SS002FRAG2_F	GTTATTAAGCCCTATCCCTAAGTCC
SS002FRAG2_R	GCCAACTTGATTAGTTAGGGAAGGC
SS002FRAG3_F	GAAAGCCACCGCCGTTGCCGTTGCC
SS002FRAG3_R	GCCAGATAACTTAATGCTTTCAGTC
SS002FRAG4_F	GATTAGGAAGATTCAGGGAGTACC
SS002FRAG4_R	CTTCAGGAAACTTGTTAACTAATTC
SS002FRAG5_F	GAAACCAATACTCTCTTAGTCGAAC
SS002FRAG5_R	GAAAAGGACCCAGGTGGCACTTTTCGGGAACGTTTACCATTGATTTCTAAC
SS002FRAGYT_F	GTTAGAAATCAATGGTAAACGTTCCCGAAAAGTGCCACCTGGGTCCTTTTC
SS002FRAGYT_R	CAACCGTGGCTCCCTCACTTTCTGGCTGTGAGTTTAGTATACATGCATTTAC
SS002FRAGBK_F	GTAAATGCATGTATACTAAACTCACAGCCAGAAAGTGAGGGAGCCACGG
SS002FRAGBK_R	GGAACTTAATTGTCATTTTAATTGGGAAGCAAAGCCAGGAAAGCGGCC
PHUPSL_US2_F	CAGGGCGCGTACTATGGTTTAGCTGTGGCTGATCCCCAGC
PHUPSL_US2_R	CGAAATATTAACAAAATCTACTTTAATAAGTATCCTGAGC
PHUPSL_PSSR2227_F	GCTCAGGATACTTATTAAAGTAGATTTTGTTAATATTTCG
PHUPSL_PSSR2227_R	GCAGCCATAAAACATTAGCCATTGATAATTTATTTCTCGTAG
PHUPSL_HUPS_F	CTACGAGAAATAAATTATCAATGGCTAATGTTTTATGGCTGC
PHUPSL_HUPL_R	GGTTATCATGAGACCGGTCTTAATTAGTACGGAACCTGGC
PHUPSL_TRRNB_F	GCCAGGTTCCGTACTAATTAAGACCGGTCTCATGATAACC
PHUPSL_SPEC_F	CAGTAATACAAGGGGTGTTATGAGGAGGATATATTTGAATAC
PHUPSL_SPEC_R1	TACAACCAATTAACCAATTCTGATTATAATTTTTTTAATCTG
PHUPSL_SPEC_R2	CCATGGTACCGCTCTGCCAGTGTTACAACCAATTAACCAATTC
PHUPSL_SPEC_R3	GGTCACTGCGGCCTGAAAAGCCATGGTACCGCTCTGCCAG
PHUPSL_DS2_F	CTGGCAGAGCGGTACCATGGCTTTTCAGGCCGCAGTGACC
PHUPSL_DS2_R	CCTGGCCTTTTGCTGGCCTTGTTTGCCGGAGAATTTTGC
PHUPSL_BONE_F	GCAAAATTCTCCGGCAAACAAGGCCAGCAAAAGGCCAGG
PHUPSL_BONE_R	GCTGGGGATCAGCCACAGCTAAACCATAGTACGCGCCCTG
PHUPW_US_F	GCGCGTACTATGGTTGTGGTGTCTATGATAGCTTC
PHUPW_US_R	GGTTTCTTAGACGTCAAACGCAGAAAGGCCCACCCG
PHUPW_PTRC_F	GGCCTTTCTGCGTTTGACGTCTAAGAAACCATTATTATC
PHUPW_PTRC_R	CTAGGTCAGTCCTCCATAAATGTGTGAAATTGTTATCCGCTC
PHUPW_HUPW_F	TTTATGGAGGACTGACCTAGATGTTAACAGTTATTGGGTGTG
PHUPW_HUPW_R1	CATTATTTATTTGAGTTATCTTTTAAATAAAAATC
PHUPW_HUPW_R2	CGCTTGCGCGGGCTTTTTCACATTATTTATTTGAGTTATC
PHUPW_HUPW_R3	TCCATAAAAAAACCCGCTTGCGCGGGCTTTTTCAC
PHUPW_HUPW_R4	AGAGTTTACCTGGCGGTTTTTCCATAAAAAAACCCGCTTG
PHUPW_DSBK_F	CAATCGAGGGGCTTTTTTTTGCCTTTTCAGGCCGCAGTGAC
PHUPW_DSBK_R	TATCATAGACACCACAACCATAGTACGCGCCCTGTAG
KOPLASMID_BB_F	AAGGCCAGCAAAAGGCCAG
KOPLASMID_BB_R	AACCATAGTACGCGCCCTGTAG
KOPLASMID_PPSBA2_F	GAAAAACCGCCAGGTAAACTC
KOPLASMID_PPSBA2_R	CCAGTGATTTTTTTCTCCATTTGGTTATAATTCCTTATGTATTTG
KOPLASMID_CM_F	ACATAAGGAATTATAACCAAATGGAGAAAAAAATCACTGG
KOPLASMID_CM_R1	GAAGCGGGTTTTTACGTTATTACGCCCCGCCCTGCCAC
KOPLASMID_CM_R2	CTCATAAAAAAACCCGCCGAAGCGGGTTTTTACGTTATTAC
KOPLASMID_CM_R3	GCAAAAAAAAGCCCCTCGATTGAGGGGCTCATAAAAAAACCCGCCG
KOPLASMID_USHESA_F	CCGCTACAGGGCGCGTACTATGGTTGTTTACGGGATGCTCATCGC
KOPLASMID_USHESA_R	AGAAGAGTTTACCTGGCGGTTTTTCGGTGAGGTGTTGAGAGTGTAC
KOPLASMID_DSHESAB_F	CCTCAATCGAGGGGCTTTTTTTTGCGGCCAAGCTACTTCTTGTAGC

(Table S3 continued)

Primer	Sequence (5′-3′)
KOPLASMID_DSHESAB_R	CGGTTCCTGGCCTTTTGCTGGCCTTCTGTGGTATGGTGCACTCTC
KOPLASMID_USHESB_F	CCGCTACAGGGCGCGTACTATGGTTAACTTCACCCCTACTGAACTC
KOPLASMID_USHESB_R	AGAAGAGTTTACCTGGCGGTTTTTCCAGCTTTTTCAGTAAGAGTGAC
KOPLASMID_USTZ_F	CCGCTACAGGGCGCGTACTATGGTTCATGTGGTCAAAGGTAGCTATC
KOPLASMID_USTZ_R	AGAAGAGTTTACCTGGCGGTTTTTCTCTTCCTCCAGTTTTTCGGGC
KOPLASMID_DST_F	CCTCAATCGAGGGGCTTTTTTTTGCTCACTTTCATAGCGTAACTC
KOPLASMID_DST_R	CGGTTCCTGGCCTTTTGCTGGCCTTTAATGGGTTAGGAGAAAGGGC
KOPLASMID_DSZ_F	CCTCAATCGAGGGGCTTTTTTTTGCCTTTGCCTCCATTTCAAAAGC
KOPLASMID_DSZ_R	CGGTTCCTGGCCTTTTGCTGGCCTTATCTTGTTTGGGATCGGTTAC
KOPLASMID_USW_F	CCGCTACAGGGCGCGTACTATGGTTGATAGCTGCTTTCTGAACTGC
KOPLASMID_USW_R	AGAAGAGTTTACCTGGCGGTTTTTCAGGGTTCTTGGGGTATCAGC
KOPLASMID_DSW_F	CCTCAATCGAGGGGCTTTTTTTTGCAACCTCCAGTCCTTTAGAAAC
KOPLASMID_DSW_R	CGGTTCCTGGCCTTTTGCTGGCCTTTGATATGGACGACGTTTGGC
KOPLASMID_USX_F	CCGCTACAGGGCGCGTACTATGGTTTCTCATTTGTCTCCTGGGGAC
KOPLASMID_USX_R	AGAAGAGTTTACCTGGCGGTTTTTCAAAGTGAGCGTTAACATGGAC
KOPLASMID_DSX_F	CCTCAATCGAGGGGCTTTTTTTTGCACTTTAAAAGGAAATCCGCC
KOPLASMID_DSX_R	CGGTTCCTGGCCTTTTGCTGGCCTTCTCAGCGAGATCGTGTAAGTC
RTPCR_NIFT_F	ATGAAAGTGATCTTAAGCAGAG
RTPCR_NIFT_R	AGGTTAGGAAATTGTAATTC
RTPCR_NIFZ_F	GTATAATCCAGGTGAAATTG
RTPCR_NIFZ_R	AACCTGAGACTCTTGTAGAG
RTPCR_NIFV_F	GATACGGTGGGCATTCTTAAC
RTPCR_NIFV_R	GGACTCTTCATGGTTTAAAC
RTPCR_NIFB_F	ATTTATCCTTGGGTTCGG
RTPCR_NIFB_R	GCGTTGGCTTTCTTTTGGGC
RTPCR_NIFS_F	CAAGTCGCATCCTTACTCGG
RTPCR_NIFS_R	AACGGGTTCCTTTACGCAC
RTPCR_NIFU_F	GAACGATTACGGAGAAAGG
RTPCR_NIFU_R	CGCTAAACAAGAGCCACATC
RTPCR_NIFH_F	GAGAATCAACTATGCGTCAG
RTPCR_NIFH_R	GTACATCGCCATCATTTCC
RTPCR_NIFD_F	GATGCTTGGTCTAGCCGTATC
RTPCR_NIFD_R	AGCCAGTGGTCCGTTTGTAG
RTPCR_NIFK_F	CTTTCTTAGGAAATGCTCGTC
RTPCR_NIFK_R	CTTTAGGAATCGGTTTGCCAG
RTPCR_NIFE_F	GAATATACCACCGCTTACGAC
RTPCR_NIFE_R	CGACCACTTCCATCCCTAAG
RTPCR_NIFN_F	GTCCTCGTGCGTCATTTTC
RTPCR_NIFN_R	GAGATACCAAACCCTTCAATG
RTPCR_HESB_F	TTATACGGGAACTCAAAC
RTPCR_HESB_R	GAAAGACTTACCGCAGCCAC
CCE_0545_RTPCR_F	GCAGCAGGTCACAATTTAG
CCE_0545_RTPCR_R	GACTACGGGGACAAGTTTC
CCE_0546_RTPCR_F	ATTTTCGTCCTCCTCGTTTCGC
CCE_0546_RTPCR_R	AAATCCAATGGTCAATCAAAAGC
CCE_0550_RTPCR_F	CAAATTCTTTGTAATTGGGTTAC
CCE_0550_RTPCR_R	TTTCAGTGCTGTCATGGAAAAC
CCE_0551_RTPCR_F	GGCTAAGAGATAAGGCAAAAC
CCE_0551_RTPCR_R	TTGGGGTATCCCAAGTGGCGGAC
CCE_0552_RTPCR_F	GTATCCCAACATCGCTCCAG
CCE_0552_RTPCR_R	GGAGTGTTTACCAACGTCTCC
CCE_0555_RTPCR_F	CTTCTTTAGTTTCTTCAACCC
CCE_0555_RTPCR_R	GTTTAGGCTAGGAAAACAACTG
CCE_0556_RTPCR_F	GTATTAACTGTTCTCGTTGTCG
CCE_0556_RTPCR_R	GTTGAAATTTCTTGGGAGTAAGC
CCE_0562_RTPCR_F	ATGGTTATGACGAGTCCTACTC
CCE_0562_RTPCR_R	GATCGACATTTTCACCACAAAC
CCE_0571_RTPCR_F	GCTACTTATCAAGTTCGCCTG
CCE_0571_RTPCR_R	CAGTTAGAACGAGGATAAGC
CCE_0572_RTPCR_F	GACAATAACGTGCTTTCTCTG
CCE_0572_RTPCR_R	TACAATTCTCCAGGGGAGAATC
CCE_0573_RTPCR_F	GAGTATCGCCATCTTGTTAAC
CCE_0573_RTPCR_R	AAAGTTCCAACCAAAGACGAGG
CCE_0574_RTPCR_F	TTCTAACAGGTTGTGGCAAAG
CCE_0574_RTPCR_R	CAATCATATTGACAGCAAGGAC
CCE_0575_RTPCR_F	CCATTGAAAGGGTGATAGAATC

(Table S3 continued)

Primer	Sequence (5′-3′)
CCE_0575_RTPCR_R	GATTAACAGAGAAATTCTCGGC
CCE_0576_RTPCR_F	GTTTACGCAAACCTTTACCATC
CCE_0576_RTPCR_R	TGTTCCTTCAACTTTCACCAC
CCE_0577_RTPCR_F	CTATTCCTATCGCCATTTTAGG
CCE_0577_RTPCR_R	GTCGGAATAATTAATTCTCCGC
CCE_0578_RTPCR_F	GTATGCTGCGGTAATTGCGGC
CCE_0578_RTPCR_R	CTCGATGGGGTTGGGTAGAAC
CCE_0579_RTPCR_F	GGAGGACACTTATTATGGC
CCE_0579_RTPCR_R	CACAGGTAAAGGGGCGTGAG
RTPCR_RNPB_F	GTTAGGGAGGGAGTTGCG
RTPCR_RNPB_R	CTGTTCCAGGATGCGAGGC
RT_Q_PCR-HUPS-F	GGCCTGTTCTGGGAATACAATTTC
RTPCR-HUPS-R	CTGGGTAAAGCTACGGAAGAAG
RTPCR-HUPL-F	AAGTGCCTCGTAATGCTATCC
RTPCR-HUPL-R	CAGGACCACCACCATACTTATC
RTPCR-HUPW-F	ACAGTTATTGGGTGTGGTAATC
RTPCR-HUPW-R	TCCCATCGAAAGTCATGTAAAC
QPCR_NIFH_F	GCGAGAATCAACTATGCG
QPCR_NIFH_R	TAGGGTCACAACCAACAATC
QPCR_NIFD_F	ACGGTGGTGTTTCTGTTC
QPCR_NIFD_R	GTTAGCGATGTGGTGTCC
QPCR_NIFK_F	GTATGGCTGAGGTTATTG
QPCR_NIFK_R	AGGTGTATGAGCGAAAGG
QPCR_RNPB_F	AAAGGTGCGGTAAGAGC
QPCR_RNPB_R	TGGGGAGTTATCTATCTGG
QPCR_CPCB_F	GCTGAAGGCAACAAACGG
QPCR_CPCB_R	ATGTCACGCAAACAAGCAGC
OripCA_Spec_F	ATGGTAAACGTTCCTAACCAGGCATCAAATAAAACGAAAG
OripCA_Spec_R1	TTTAATATTTAAGCTCCATGGTACCGCTCTGCCAG
OripCA_Spec_R2	AGAAATATTAAATAATTTAATATTTAAGCTCCATGG
OripCA_pca2.4_F	AGCTTAAATATTAAATTATTTAATATTTC
OripCA_DS_F2	GCTATTTTCTTAATTGAGTAAGTCTATTACAATTAAAATG
OripCA_pca2.4_R	TAATAGACTTACTCAATTAAGAAAATAGC
OripCA_DS_F1	TTGAGTAAGTCTATTACAATTAAAATGACAATTAAGTTCC
OripCA_DS_R1	CTTTTGCTGGCCTTGAATTCATGAGGTAGGTTGGAAACGC
OripCA_DS_R2	TTTACGGTTCCTGGCCTTTTGCTGGCCTTGAATTC
OripCA_US_F2	ATGCGCCGCTACAGGGCGCGTACTATGGTTGGATCCCCTGAC
OripCA_US_F1	TACTATGGTTGGATCCCCTGACATCGAAGTCGAAGC
OripCA_spec_R3	AATACCAGAAACTCCCCATGGTACCGCTCTGCCAG
OripCA_pcB2.4_F	AGAGCGGTACCATGGGGAGTTTCTGGTATTTTCTAGC
OripCA_pcB2.4_R	CAAATTTGACTAATAGTTTTGATATTTTATTAC
OripCA_DS_F4	TAAAATATCAAAACTATTAGTCAAATTTGCAATTAAAATG
OripCA_DS_F3	TATTAGTCAAATTTGCAATTAAAATGACAATTAAGTTC
Orichange_pCCSpec_R4	GCTTTAATAATCTCGCCATGGTACCGCTCTGCCAG
Orichange_pCC5.2_F	AGAGCGGTACCATGGCGAGATTATTAAAGCCAAGACGG
Orichange_pCC5.2middle_R	CGGGAGTACCCTAGGGTAGTC
Orichange_pCC5.2middle_F	CAGGGCATTATTGACCGCTAC
Orichange_pCC5.2_R	TTGTCATTTTAATTGCCTTATCAGTCTTGGTAGCGATC
Orichange_pCCDS_F5	CCAAGACTGATAAGGCAATTAAAATGACAATTAAGTTCCTGTC
Orichange_pCCDS_R3	CCTTTTGCTGGCCTTGTCGACATGAGGTAGGTTGGAAACGC
Orichange_pCCDS_R4	TTTTACGGTTCCTGGCCTTTTGCTGGCCTTGTCGAC
