# Supplementary material for: Engineering Nitrogen Fixation Activity in an Oxygenic Phototroph
Source: mBio. 2018 Jun 5;9(3):e01029-18. doi: 10.1128/mBio.01029-18 (PMC5989072; doi:10.1128/mBio.01029-18)
Supplement: TABLE S1 [file mbo003183927st1.pdf]

Table S1 Impact of various *nif*-gene inactivations on nitrogenase activity in different engineered strains. Relative activities with respect to that for the respective engineered strain with wild-type *nif* cluster.

Genotype	*E. coli*[a]	*E. coli*[b]	*Synechocystis* 6803[c]
wild-type cluster	100%	100%	100%
Δ*nifT*	-	-	4%
Δ*nifV*	1%	25%	-
Δ*nifB*	0%	0%	-
Δ*nifS*	5%	-	-
Δ*nifU*	10%	-	-
Δ*nifX*	-	50%	<1%
Δ*nifW*	-	-	7%
Δ*hesA*	-	50%	-
Δ*hesB*	-	-	9%
Δ*nifQ*	90%	-	-
Δ*nifF*	50%	-	-
Δ*nifJ*	30%	-	-
Δ*nifTZ*	-	-	<1%
Δ*hesAB*	-	-	6%
Δ*nifXhesA*	-	35%	-
Δ*nifVhesA*	-	25%	-
Δ*nifVXhesA*	-	7%	-
Δ*nifVXNhesA*	-	2%	-
Δ*nifENX*	110%	-	-
Δ*nifUSV*	0%	-	-
Δ*nifWZM*	100%	-	-

[a]Nitrogen fixation genes from *A. vinelandii* and *K. oxytoca*. Data from (1).

[b]Nitrogen fixation genes from *Paenibacillus*. Data from (2).

[c]Activities shown here are based on the strain TSyNif-2 with 100% activity (this study)
